# BA.2 and BA.5 omicron differ immunologically from both BA.1 omicron and pre-omicron variants

**DOI:** 10.1038/s41467-022-35312-3

**Published:** 2022-12-13

**Authors:** Annika Rössler, Antonia Netzl, Ludwig Knabl, Helena Schäfer, Samuel H. Wilks, David Bante, Barbara Falkensammer, Wegene Borena, Dorothee von Laer, Derek J. Smith, Janine Kimpel

**Affiliations:** 1 Institute of Virology, Department of Hygiene, Microbiology and Public Health, Medical University of Innsbruck, Peter-Mayr-Str. 4b, 6020 Innsbruck, Austria; 2 University of Cambridge, Center for Pathogen Evolution, Department of Zoology, Cambridge, UK; 3 Tyrolpath Obrist Brunhuber GmbH, Hauptplatz 4, 6511 Zams, Austria

**Keywords:** Epidemiology, Respiratory tract diseases, Viral infection, SARS-CoV-2, Viral immune evasion

## Abstract

Several studies have shown that SARS-CoV-2 BA.1 omicron is an immune escape variant. Meanwhile, however, omicron BA.2 and BA.5 became dominant in many countries and replaced BA.1. As both have several mutations compared to BA.1, we analyzed whether BA.2 and BA.5 show further immune escape relative to BA.1. Here, we characterized neutralization profiles against the BA.2 and BA.5 omicron sub-variants in plasma samples from individuals with different history of exposures to infection/vaccination and found that unvaccinated individuals after a single exposure to BA.2 had limited cross-neutralizing antibodies to pre-omicron variants and to BA.1. Consequently, our antigenic map including all Variants of Concern and BA.1, BA.2 and BA.5 omicron sub-variants, showed that all omicron sub-variants are distinct to pre-omicron variants, but that the three omicron variants are also antigenically distinct from each other. The antibody landscapes illustrate that cross-neutralizing antibodies against the current antigenic space, as described in our maps, are generated only after three or more exposures to antigenically close variants but also after two exposures to antigenically distant variants. Here, we describe the antigenic space inhabited by the relevant SARS-CoV-2 variants, the understanding of which will have important implications for further vaccine strain adaptations.

## Abstract (web summary)

SARS-CoV-2 omicron variant BA.1 has shown increased transmissibility and immune escape, relative to previous SARS-CoV-2 variants. In this study, authors utilise antigenic cartography to characterise the neutralisation profiles of omicron sub-lineages, BA.2 and BA.5, in comparison to BA.1 and pre-omicron variants.

## Introduction

During the course of the severe acute respiratory syndrome coronavirus-2 (SARS-CoV-2) pandemic, mutations in the viral genome occurred leading to an evolution from ancestral variants to currently circulating Variants of Concern (VoC). Variants that were selected either had improved viral fitness/transmission kinetics such as the alpha (B.1.1.7) variant, immune escape properties, such as the beta (B.1.351) variant or a combination of both such as the delta (B.1.617.2) variant. Most recently, the omicron (B.1.1.529) variant has been described as VoC with several sub-lineages, including BA.1, BA.2, BA.4, BA.5, and BA.2.12.1, of which BA.5 recently became dominant in many countries.

We and others previously showed that the BA.1 omicron variant led to the strongest immune escape so far seen in SARS-CoV-2 variants^1–4^. While the virus strongly escapes neutralizing antibodies induced by infection with pre-omicron variants or two doses of vaccination, T cell responses seem to be more conserved^5–7^. However, multiple exposures improve neutralizing antibody titers against BA.1 omicron as seen in individuals after booster vaccination or hybrid immunity^1,2,8,9^.

BA.1, BA.2, and BA.5 omicron sub-variants share common mutations; however, each also has unique mutations. Some of these unique mutations are located in the receptor-binding domain or the N-terminal domain of the spike protein, both important epitopes for the binding of neutralizing antibodies. Therefore, the omicron sub-variants might have distinct neutralization profiles. The antigenic difference of pre-omicron, BA.1 omicron and BA.2 omicron variants is supported by a study showing distinct profiles of sensitivity against therapeutic monoclonal antibodies^10^. Initial reports analyzing sera from wild-type convalescent or two-dose vaccinated individuals show that both omicron subvariants escape neutralizing antibody responses to a similar degree perhaps with a trend of lower escape by BA.2. A booster immunization or hybrid immunity strongly enhances neutralizing antibodies against both omicron variants^11–13^. However, these studies mainly analyzed samples from vaccinated individuals and little is known about neutralizing antibody profiles induced by BA.2 omicron infection in previously naïve individuals.

Antigenic cartography is a tool to visualize antigenic differences between different virus variants. Several maps have been described showing pre-omicron variants and also including the BA.1 omicron variant^14–17^. The position of the BA.2 omicron variant has so far been only added to maps using sera from infected hamsters but no human data are available^18^.

In the current study, we characterize neutralization profiles against the new BA.2 and BA.5 omicron variants in plasma samples from a variety of individuals with different numbers of exposures to infection/vaccination, including samples from previously naïve BA.2 infected individuals, and use these data to generate an antigenic map including all current VoC.

## Results

**Fig. 1:**
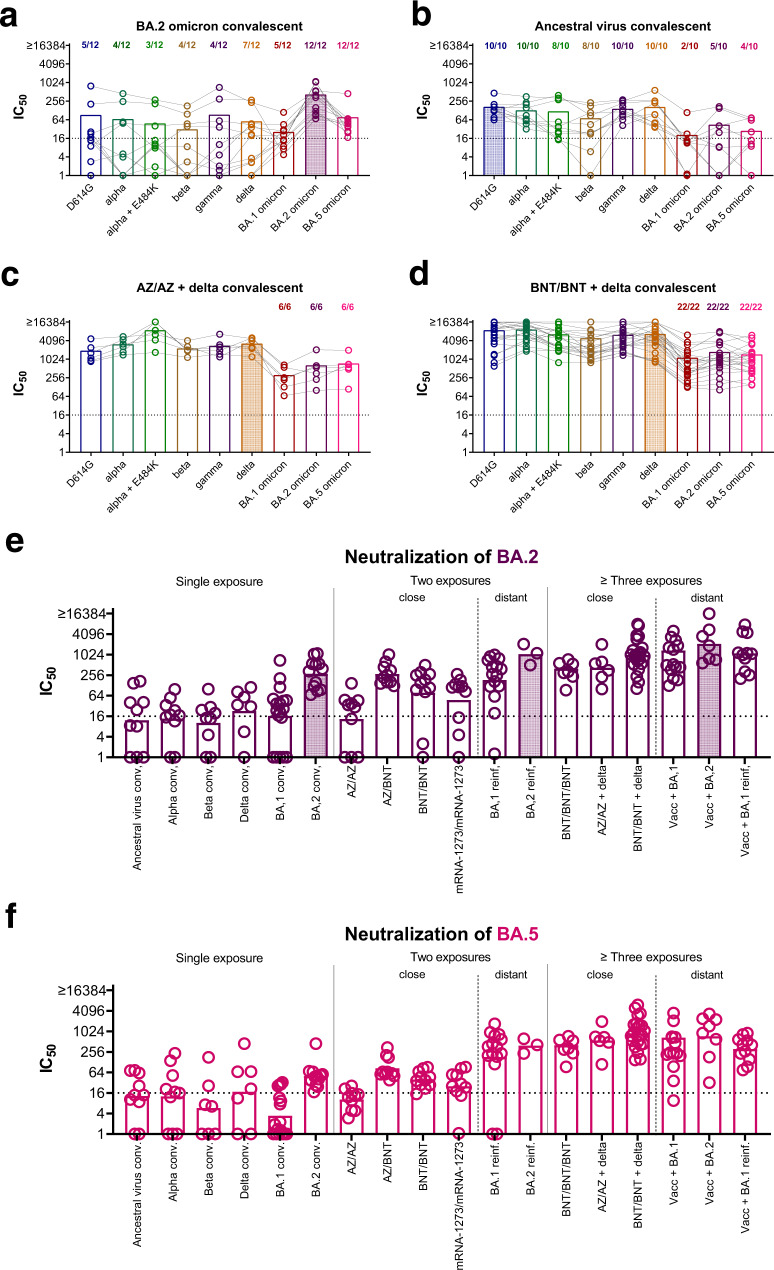
BA.2 and BA.5 omicron have a neutralization profile distinct from both pre-omicron variants and BA.1 omicron. Plasma samples were collected after a BA.2 omicron (*n* = 12, Panel **a**) or wild-type infection (*n* = 10, Panel **b**) in previously naïve patients (no vaccination and no known history of prior infection) or in patients with hybrid immunity, Panel **c** two doses of ChAdOx-S1 (AZ/AZ) followed by delta infection (*n* = 6) and Panel **d** two doses of BNT162b2 (BNT/BNT) followed by pre-omicron variant (presumably delta) infection (*n* = 22). Samples were analyzed for 50 % neutralizing antibody titers (IC_50_) against D614G (blue), alpha (dark green), alpha + E484K (light green), beta (ocher), gamma (purple), delta (orange), BA.1 (red), BA.2 (violet), and BA.5 (pink) omicron. Shown are individual patient samples as circles connected by lines and mean titers as bars. Numbers above bars indicate proportion of positive samples (titers > 1:16). IC_50_ titers against BA.2 omicron (Panel **e**; violet) and BA.5 omicron (Panel **f**; pink) for different groups of individuals with single exposure (non-vaccinated convalescent: ancestral virus conv. *n* = 10; alpha conv. *n* = 10; beta conv. *n* = 9; delta conv. *n* = 7; BA.1 conv. *n* = 18; BA.2 conv. *n* = 12), two exposures with close variants (two doses of vaccine: ChAdOx-S1/ChAdOx-S1 (AZ/AZ) *n* = 10; ChAdOx-S1/BNT162b2 (AZ/BNT) *n* = 10; BNT162b2/BNT162b2 (BNT/BNT) *n* = 11; mRNA-1273/mRNA-1273 *n* = 10) two exposures with distant variants (re-infection with BA.1 (*n* = 15) or BA.2 omicron (*n* = 3) after historic non-omicron infection in unvaccinated), three or more exposures with close variants (three doses of vaccination (BNT162b2/BNT162b2/BNT162b2 (BNT/BNT/BNT) *n* = 7) or breakthrough infection with delta variant after vaccination (AZ/AZ + delta *n* = 6; BNT/BNT + delta *n* = 22)), and three or more exposures with distant variants (breakthrough infection or reinfection with a omicron variant after vaccination (Vacc + BA.1 *n* = 14; Vacc+BA.2 *n* = 7; Vacc+BA.1 reinfected *n* = 11)) were determined. D614G, alpha, beta, gamma, delta, and BA.1 omicron IC_50_ for these groups have been published previously in^1,2^. Shown are individual patients (circles) and geometric mean (bars) for each group. The dotted lines in panels **a**–**f** indicate the limit of detection.

First, we analyzed neutralizing antibody profiles in previously naïve individuals after infection with the BA.2 omicron variant. All of these individuals had detectable neutralizing antibodies against the BA.2 omicron variant itself and also BA.5 omicron, although with lower titers, however neutralizing antibodies against pre-omicron and BA.1 omicron were only occasionally above the limit of detection (IC_50_ > 1:16) and in the few positive samples generally low (Fig. 1a). This was in concordance with our previous data where unvaccinated individuals after a pre-omicron VoC infection induced mainly neutralizing antibodies against pre-omicron variants but not BA.1 omicron and vice versa, sera from unvaccinated individuals recovered from BA.1 omicron variant infection mainly neutralized BA.1 omicron but not pre-omicron variants^1,2^.

To complete the dataset, we now also analyzed neutralizing antibody titers against our panel of variants for plasma samples from individuals who had been infected during the first wave in Austria (March/April 2020) with an ancestral virus variant^19^. As expected, these individuals had high neutralizing antibody titers against pre-omicron variants with slightly reduced titers against the immune escape variants beta and alpha with E484K mutation (for both variants 8 out of 10 individuals above cut-off). However, neutralizing antibodies against all three omicron sub-variants were only induced in part of the individuals (2 out of 10 for BA.1, 5 out of 10 for BA.2, and 4 out of 10 for BA.5, Fig. 1b). In contrast, individuals with hybrid immunity showed a broad neutralizing antibody response against all variants analyzed (Fig. 1c, d). This was true for breakthrough infections after two doses of an mRNA vaccination (BNT162b2, BNT) as well as after two doses with a vector vaccine (ChAxOx-1-S, AZ). These breakthrough infections were presumably all caused by the delta variant.

We next analyzed neutralizing antibody titers against the BA.2 and BA.5 omicron variants for a broader selection of samples. We included individuals with single exposure (unvaccinated convalescent from ancestral virus, alpha, beta, delta, BA.1 omicron, or BA.2 omicron variant), with two close exposures (vaccinated with two doses), two distant exposures (unvaccinated after pre-omicron variant and BA.1 or BA.2 omicron re-infection), three and more close exposures (vaccinated with three doses or vaccinated with two/three doses and pre-omicron breakthrough infection) or three and more distant exposures (vaccinated with two or three doses and breakthrough infection with an omicron variant). In general, multiple exposures improved neutralizing antibody titers against the BA.2 and BA.5 omicron variants even for individuals that had had no contact with the BA.2 or BA.5 omicron variant itself (Fig. 1e+f, Tables S2+S3).

**Fig. 2:**
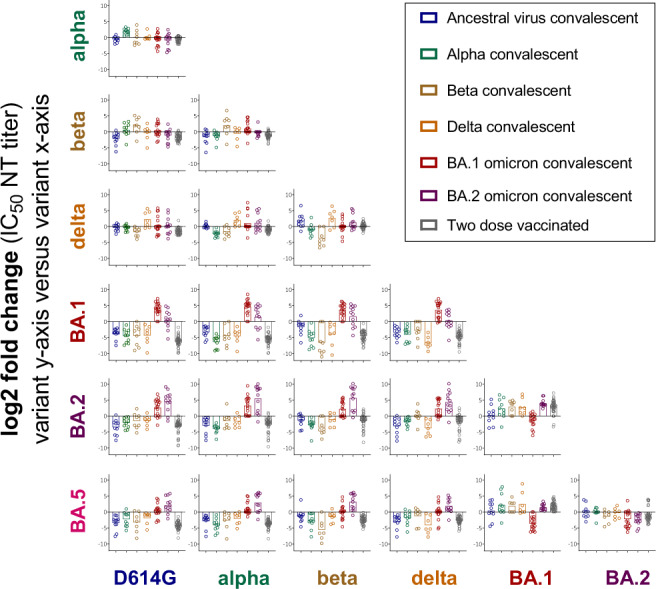
Fold-change of neutralizing antibody titers between variant depicted on y-axis and variant on x-axis. For each single exposure (unvaccinated ancestral *n* = 10, alpha *n* = 10, beta *n* = 8, delta *n* = 7, BA.1 omicron *n* = 18, or BA.2 omicron *n* = 12 convalescent individuals) or two-dose vaccinated serum (*n* = 41) the fold change of neutralizing antibody titers for the different variants was calculated. Part of the neutralizing antibody titers used for the calculation of fold changes have been previously published in^1,2^. Shown are individual patients (circles) and geometric mean fold changes (bars).

We next calculated relative changes in neutralizing antibody titers between different pre-omicron (D614G, alpha, beta delta) and omicron (BA.1, BA.2, BA.5) variants in our single exposure and two-dose vaccinated cohorts based on previously published data^1,2^ and the data presented in Fig. 1. Single exposure convalescent sera were best neutralized by the exposed variant or similar variants (Fig. 2). Sera from two doses vaccinated individuals behaved similar as single exposure sera from pre-omicron variant convalescents. Titers to more diverse variants dropped in some cases more than 10-fold, indicating different serotypes. Relative to pre-omicron variants the three omicron variants showed increasing immune escape in the order BA.2<BA.5<BA.1.

**Fig. 3:**
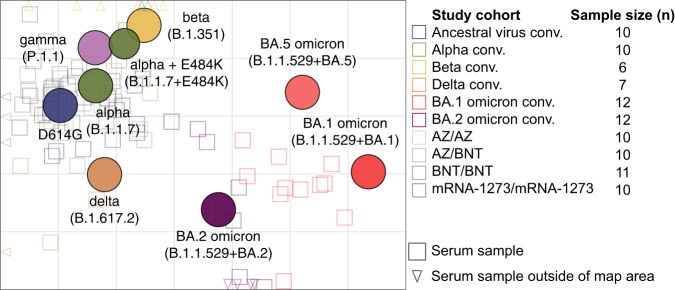
Antigenic map of SARS-CoV-2 variants constructed from single exposure convalescent and double vaccinated sera. Virus variants are shown as colored circles, sera as open squares with the color corresponding to the infecting variant. Triangles indicate sera positioned outside the central map area and point in the direction where the corresponding serum is positioned. Vaccine sera are shown in grey tones (from dark to light: mRNA-1273/mRNA-1273 *n* = 10, BNT162b2/BNT162b2 *(BNT/BNT) n* = 11, ChAdOx-S1/BNT162b2 (AZ/BNT) *n* = 10, ChAdOx-S1/ChAdOx-S1 (AZ/AZ) *n* = 10). The alpha + E484K variant is shown as smaller circle due to its additional substitution compared to the alpha variant. The x- and y-axis represent antigenic distances with one grid square corresponding to one two-fold serum dilution of the neutralization titer. The map orientation within x- and y-axis is free as only relative distances can be inferred. A non-zoomed in version of the map is shown in Figure S2. Only single-variant exposure sera have been used for construction of the map. *n* = 2 beta convalescent and *n* = 6 BA.1 convalescent samples could not be positioned in the map because of too many <LOD titers. The table in the right part indicates how many sera from each group were positioned on the map (Ancestral virus conv. *n* = 10, Alpha conv. *n* = 10, Beta conv. *n* = 6, Delta conv. *n* = 7, BA.1 omicron conv. *n* = 12, BA.2 omicron conv. *n* = 12). Each of these serum samples has been titrated against D614G, alpha, alpha E484K, beta, gamma, delta, BA.1 omicron, BA.2 omicron, and BA.5 omicron variants. Neutralizing antibody titers are shown in Fig. 1 or have been published previously in^1,2^.

These data indicate that BA.2 and BA.5 omicron variants are positioned antigenically between pre-omicron variants and the BA.1 omicron variant but distinct to both and distinct to each other. To analyze these differences in more detail, we next did antigenic cartography, for which a matrix of neutralizing antibody titers against different virus variants for a panel of single variant exposure sera is used to obtain map distances^20^. Antigen variants and sera are positioned relative to each other in a lower-dimensional space based on fold changes of serum antibody titers from exposed variant to other variants, where the serum-antigen distance in the map reflects the dilution steps in the neutralization assay (Supplementary Methods Map construction). Applying this approach to single infection or two dose vaccinated sera, as we did here, allows to estimate antigenic relationships of SARS-CoV-2 variants, whereas multi-exposure sera would underestimate the underlying differences between variants due to increased cross-reactivity and result in smaller map distances between variants. The variation we observed in the titer data was mostly due to variation in titer point estimates that we extrapolated below the limit of detection. These point estimates were not used in the cartography, but treated as less than the limit of detection as described in the methods section. The map represents the titer data from convalescent and double vaccinated serum groups well in 2D and was robust to assay noise and sample size (Fig. 3, Figs. S2–S10, S14–18 and Supplementary Methods). Figure 3 shows the central part of the map only with the number of sera from each group used for the construction of the map indicated in the right part, while Fig. S2 represents a non-zoomed version. There is a good correspondence of sera and antigen map position.

The antigenic map visualizes the substantial difference of the three omicron sub-lineages BA.1, BA.2 and BA.5 compared to the previously circulating variants (Fig. 3) and corresponds well with previous maps^14,15,18,21^. Wu-1 like variants (D614G, alpha, alpha+E484K), beta and gamma occupy a small space in the map and the delta variant is in roughly the same area. Barely detectable neutralization titers against BA.1 omicron in all but the BA.1 omicron convalescent serum group resulted in its positioning far away from the other variants. As the titer data indicated, BA.2 and BA.5 omicron were located between the pre-omicron variants and BA.1 omicron, with BA.2 approximately equidistant to delta and BA.1 omicron. Interestingly, the distance of BA.2 to delta is smaller than the delta-beta distance. This can be explained by similar titer drops to delta and BA.2 in the beta convalescents, only marginally higher drops to BA.2 than to beta in the delta convalescents, and higher delta than beta titers in the BA.2 convalescents (Fig. 2).

Despite higher BA.5 titers in the BA.2 convalescent samples, its map position is closer to BA.1 than BA.2. We explain this by the pronounced drop of titers from BA.2 to BA.5 in the BA.2 omicron convalescent group and the lower BA.5 than BA.2 titers in the BA.1 omicron convalescents, pushing BA.5 away from BA.2 (Figure S7). We found two different patterns in the BA.2 convalescent sera, one with high cross-reactivity and the other with little. This resulted in two serum clusters in the map, a central one for the cross-reactive and far-off positions for BA.2 specific sera (Fig. 1a, Figure S2, Figure S11). The high cross-reactivity could be due to undetected prior infection. Particularly two of the 12 BA.2 omicron convalescent sera had a broadly cross-reactive pattern, which could be explained by a previous undetected infection (Fig. 1a). A potential issue with identifying single-infection sera from humans and consequently a weakness of our study is that these persons might have had a previous asymptomatic infection that have altered their reactivity profile and might thus not accurately represent the basic antigenic relationships among variants. To control for that, we created a map excluding the two BA.2 convalescent sera with high cross-reactivity and a potential previous exposure. However, this did not impact map conformation (Figure S12).

**Fig. 4:**
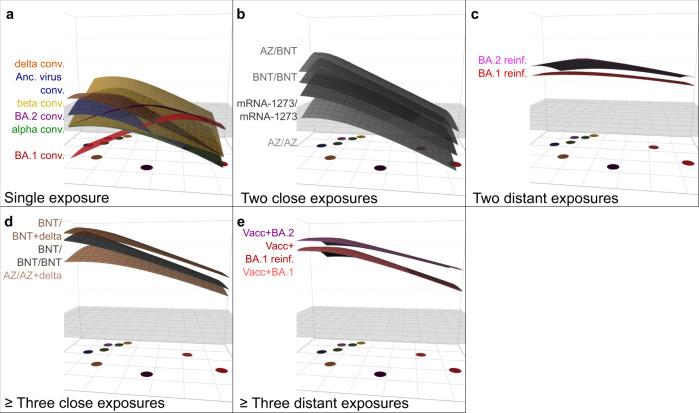
GMT antibody landscapes by exposure history. The colored surfaces represent the GMT antibody landscapes of the different serum groups. The map shown in Fig. 3 serves as base plane, the z-axis reflects log_2_ titers with each two-fold increase marked starting from titer 20. The grey plane at titer 50 serves as reference. The GMT antibody landscapes were grouped and colored by virus exposures: Panel **a**. Single exposure convalescent sera (from top to bottom: delta (orange), ancestral virus (blue), beta (yellow), BA.2 omicron (purple), alpha (green), and BA.1 omicron (red)). Panel **b**. Two close exposures (from top to bottom: ChAdOx-S1/BNT162b2 (AZ/BNT), BNT162b2/BNT162b2 (BNT/BNT), mRNA-1273/mRNA-1273, ChAdOx-S1/ChAdOx-S1 (AZ/AZ)). Panel **c**. Two distant exposures (from top to bottom: pre-omicron infection followed by BA.2 omicron reinfection (BA.2 reinf., pink), pre-omicron infection followed by BA.2 omicron reinfection (BA.1 reinf., red)). Panel **d**. Three or more close exposures (from top to bottom: BNT/BNT + delta breakthrough (BNT/BNT + delta, light brown), three dose BNT vaccination (BNT/BNT/BNT, dark grey), AZ/AZ + delta breakthrough (AZ/AZ + delta, orange)). Panel **e**. Three or more distant exposures (from top to bottom: Vacc + BA.2 omicron breakthrough (Vacc + BA.2, purple), Vacc + BA.1 reinfection (Vacc + pre-omicron breakthrough infection + BA.1 omicron reinfection, red), Vacc + BA.1 omicron breakthrough (Vacc + BA.1, salmon)). Individual sera antibody landscapes are shown in Fig. S11.

To visualize the antibody reactivity profile of individuals with distinct infection history, we next constructed antibody landscapes^14,22^ for each serum group (see Fig. 4 for GMT landscapes and Figure S11 for individual landscapes). Using an antigenic map from first infection and two dose vaccinated sera as the basis for the variants’ antigenic relationships, antigenic landscapes show quantitatively how titer magnitude and breadth distribute over the mapped strains. The base map sets the variants’ x- and y- coordinates in antigenic space, and the measured neutralization titer against a variant gives the height of the landscape in the z-direction. This methodology allows analyzing complex serological data beyond primary exposures, as more cross-reactive sera will have flatter landscapes than strain-specific first-exposure sera. While this change in cross-neutralization could be visualized in an antigenic map with variants occupying a smaller space^23^, antibody landscapes are necessary to examine titer magnitude and distribution over mapped space. Grouping by exposure history, we found that at least two distant variant encounters increased cross-reactivity of neutralizing antibodies to other variants. Single-variant exposure landscapes had highest reactivity against the infected variant with little cross-reactivity to antigenically distant variants. Ancestral virus, delta convalescents and two dose vaccinated individuals or alpha and beta convalescents shared similar reactivity profiles (Fig. 4a–b, Figure S11A–J). BA.1 and BA.2 omicron convalescent landscapes exhibited both unique antibody profiles focused on the area of their root variant, reflecting the observations in the neutralizing antibody titer data.

In contrast, non-omicron infection followed by omicron infection resulted in broad antibody reactivity profiles of similar shape, differing only in magnitude by omicron sub-lineage (Fig. 4c). Similarly, triple vaccination and breakthrough infections resulted in broad GMT antibody landscapes of similar shape (Fig. 4d, e). Looking at individual landscapes, we found that delta breakthrough infections elicited slightly higher titers against BA.2 and BA.5 omicron than triple vaccination (Fig. S11M–O). BA.1 omicron breakthrough and BA.1 omicron breakthrough with previous non-omicron infection landscapes were almost identical (Fig. 4e, S11P, R). This suggests that, while the increase in cross-reactivity from one to two SARS-CoV-2 variant exposures is substantial, a third variant exposure changes the reactivity profile not to the same extent. Examining this in more detail, we found that the number of exposures increased antibody levels, but the type of exposure and variant impacted the landscape’s shape (Fig. S13). On average, antibody reactivity in the multi-exposure cohorts was higher against pre-omicron variants than against the omicron sub-lineages with the exception of omicron re-infected individuals.

## Discussion

Our results support the hypothesis that the omicron variant is indeed a distinct serotype compared to previous VoC. However, we found the three omicron sub-lineages BA.1, BA.2 and BA.5 to be antigenically distinct both from each other and from pre-omicron variants resulting in different neutralization profiles and distinct positions on the antigenic map.

The most basic antigenic relationships among variants are measured using sera from primary-infections with a single variant. We also considered two doses of monovalent ancestral variant vaccine as single variant exposure sera and used them for generation of the antigenic map as they give sufficiently similar fold-drops to convalescent primary-infection sera (Fig. 2, Fig. S11A, G–J)^9^. Such primary infection sera will become increasingly more difficult to acquire as completely naïve individuals without a history of infection or vaccination become rare. This highlights the importance of animal model sera to study the basic SARS-CoV-2 antigenic relationships, similar to that which has been established for influenza for decades^20^.

Sera occupying different areas of a map increase the triangulation of individual points (see Supplementary Materials Map resolution). To accurately estimate the similarity of omicron sub-lineages, it is thus vital to construct a map including omicron first infection sera, which are the only sera resolving the lower right map area. To our knowledge, the data and cartography we present is unique in having both BA.1 and BA.2 primary infection sera for the resolution of the basic antigenic relationship among BA.1, BA.2 and BA.5 omicron variants based on data from human sera using replication-competent SARS-CoV-2 variant-based assays. The antigenic map we present here and those by others showed the three sub-lineages as antigenically distinct from pre-omicron VoCs and each other, the location of pre-omicron VoCs correspond well between maps^14,15,23,24^. Differences of BA.2 and BA.5 positions relative to the other VoCs might be explained by maps constructed from different serum groups, adding or decreasing map resolution in different areas of antigenic space (Supplementary Materials Map resolution). Wang et al. did not include BA.2 convalescent sera in their map, which might explain the proximity of BA.2 and BA.4/5 in their map^23^. In the map we present here and that by van der Straten et al., BA.2 appears antigenically more similar to delta than delta to beta^15^. Liu et al. and Cele et al. have reported substantial escape of the beta variant from delta convalescent sera^25,26^. Liu et al. attribute this to the differences of the two variants at positions 417, 484, and 501^25^. While BA.2 shares these substitutions with beta and not with delta, BA.2 and delta have mutation T478K in common which could contribute to detectable delta titers in the BA.2 convalescents and vice versa^23^. Wilks et al. have shown that different sera exhibit distinct sensitivities to certain spike mutations^14^.

To compare magnitude and breadth of neutralization titers in complex serological data we used antibody landscapes. Looking at how neutralizing antibody reactivity distributed across the mapped antigenic space through antibody landscapes, individuals exposed to a single variant exhibited mainly titers against the specific or closely related variants. A subsequent infection with an antigenically distant strain did not only increase titers against the new variant, but also against the previous variant and resulted in broad reactivity profiles even to variants the individual was not exposed to (Fig. 4c). This is in line with other studies where an omicron variant re-infection not only increased neutralizing antibody titers against omicron but also against other variants^27,28^. Surprisingly, we found that a delta variant breakthrough infection induced high titers against the omicron sub-lineages, although these individuals were never exposed to an omicron variant. In these individuals, neutralizing antibody titers against the omicron sub-variants were comparable to individuals who had a combination of prior infection/vaccination and omicron infection. We found that triple vaccination also increased titers against omicron variants, suggesting similar cross-reactivity of the antibody repertoire after two distant infections and triple vaccination. One limitation of our study is that most samples were collected shortly after infection, presumably during the peak of the responses, and therefore we could not analyze longevity of cross-neutralizing antibodies. However, others also found improved levels and durability of cross-neutralizing antibodies after three or more exposures (vaccination or infection)^3,8,28^. A second limitation of our study is that exposure history of study participants could be complicated by previous undetected asymptomatic infections. However, removing sera with a potential previous exposure history, i.e. cross-reactive sera from the BA.2 convalescent group, did not change our map indicating that primary infection sera were the main determinants of map topology and hence antigenic relationships. In a non-human primate model, titers of cross-neutralizing antibodies were similarly increased by either a third dose of a wild type-specific mRNA vaccine or a beta-specific mRNA vaccine after two doses of a wild type-specific mRNA vaccine^29^.

Others have shown that the antibody and memory B cell repertoire is comparable or even superior after infection followed by a single vaccine dose compared to double vaccination only^30–33^. We report here that the immune profile after triple vaccination is very similar to the profile of two doses followed by delta breakthrough, finding only marginally better cross-neutralization after breakthrough than vaccination (Fig. 4, Figure S11). Based on the similarity of delta breakthrough and triple vaccine landscape, we suggest that a vaccine update including an omicron variant would induce antibody neutralization profiles comparable to omicron breakthrough infections. Indeed, recent clinical studies have shown the potential of omicron-containing vaccines to boost titers across antigenic space^34–36^.

We propose three hypotheses for the improved cross-neutralization after repeated exposure, even for non-exposed variants. Firstly, this could be a consequence of antibody saturation against the encountered virus variants while the reactivity against unencountered viruses could still be boosted with overall increasing antibody titers, resulting in smaller differences in neutralization between exposed and unexposed variants with overall increased antibody titers. Secondly, the prior encounter of two different pre-omicron variants could boost immunity against conserved epitopes shared with the omicron sub-lineages. Some monoclonal antibodies have been shown to retain activity against BA.1 and/or BA.2 omicron^37^. Thirdly, the broad polyclonality of the response after exposure to two different variants might contribute to omicron neutralization, as a cocktail of monoclonal antibodies was reported to improve neutralization of BA.1 and BA.2 omicron^10,38^.

Our work presents an important contribution to the discussion on vaccine updates to improve protection against current and future emerging SARS-CoV-2 variants. It suggests, that cross-neutralization improves with repeated exposure and an increase in absolute titers of antibodies and that exposure to two distant variants has protective potential against emerging variants with some degree of similarity to currently and previously circulating VoCs.

## Methods

### Ethics statement

The ethics committee (EC) of the Medical University of Innsbruck has approved the study with EC numbers: 1100/2020, 1111/2020, 1330/2020, 1064/2021, 1093/2021, 1168/2021, 1191/2021, and 1197/2021. Informed consent has been obtained from study participants.

### Patient characteristics

We included individuals with single, double or three and more exposures. Details regarding age, sex, vaccination status etc. of participants are given in the Supplementary Methods and Table S1.

### Neutralization assay

A focus forming neutralization assay was performed to determine neutralization titers against various SARS-CoV-2 variants^1,39^. Briefly, four-fold serial dilutions (1:16 to 1:16,384) of heat-inactivated serum samples were pre-incubated with SARS-CoV-2 virus isolates for 1 h and subsequently used to infect 90% confluent Vero cells stably overexpressing TMPRSS2 and ACE2. After two hours supernatant was replaced by fresh medium and cells were fixed further 8 h later with 96% EtOH for 5–10 min. Infected cells were stained using a SARS-CoV-2 convalescent serum (1:1000 diluted) and a polyclonal goat anti-Human Alexa Fluor Plus 488-conjugated goat anti-human IgG secondary antibody (1:1000 diluted; Ref. A48276, Invitrogen, Thermo Fisher Scientific, Vienna, Austria) and counted using an immunospot reader. Continuous 50% neutralization titers were calculated in GraphPad Prism 9.0.1 (GraphPad Software, Inc., La Jolla, CA, USA) using a non-linear regression. Titers <1:16 were considered negative. Titers <1 were set to 1 and titers >1:16,384 were set to 1:16,384. The following replication competent SARS-CoV-2 virus isolates have been used: D614G (Isolate B86.2, GISAID ID EPI_ISL_3305837); alpha variant (B.1.1.7, isolate C69.1, GISAID ID EPI_ISL_3277382); alpha variant with E484K mutation (C79.2, GISAID ID EPI_ISL_3277383); beta variant (B.1.351, isolate C24.1, GISAID ID EPI_ISL_1123262); gamma variant (P.1.1, isolate hCoV-19/Germany/BY-MVP-000005870/2021, GISAID ID EPI_ISL_2095177); delta variant (B.1.617.2, isolate SARS-CoV-2-hCoV-19/USA/NY-MSHSPSP-PV29995/2021, GISAID ID EPI_ISL_2290769); BA.1 omicron variant (isolate E16.1, GISAID ID EPI_ISL_6902053); BA.2 omicron variant (isolate E65.1, GISAID ID EPI_ISL_12486408); BA.5.3.2 omicron variant (BA.5-like, isolate E73.1, GISAID ID EPI_ISL_13666092). Viruses were grown on Vero cells stably overexpressing TMPRSS2 and ACE2. Cells had been generated in-house for a previous study^39^.

### Antigenic cartography

Antigenic maps were constructed from single infection and double vaccination sera as described in^14,20^. Using multidimensional scaling, antigen variants and sera are positioned in a lower-dimensional space based on serum antibody titers. For each serum and antigen pair, antigenic distances are calculated from the titer reduction of the antigen against which the specific serum has the highest titer (usually the homologous, infecting antigen) to antigen i. For each serum-antigen pair, serum and antigen coordinates are optimized to minimize the error between the Euclidean distance in the map and the target distance from the measured titers, where one antigenic unit in the map corresponds to one two-fold dilution of titers in the neutralization assay. A more detailed description of the algorithm can be found in the supplementary methods. R’s (Version 4.2.0) “Racmacs” package^40^ (Version 1.1.35) was used to create antigenic maps with 1000 optimizations, a dilution stepsize of 0, the minimum column basis set to “none”, and titers below the limit of detection of 16 set to “<16”, which results in penalization of map distances shorter than the target distance but no penalty for greater map than target distances. *N* = 2 beta convalescent and *n* = 6 BA.1 convalescent samples could not be positioned in the map because of too many <LOD titers. The reactivity of the P.1.1 variant was reduced by one two-fold due to as high or higher than homologous titers in all but the BA.1 and BA.2 convalescent serum groups (Fig. S1). A two-dimensional map was suitable to represent the antigenic relationships as assessed by map diagnostics (Supplementary Methods).

### Antibody landscapes

First infection and double vaccination antibody landscapes were constructed as described in^14^ using the P.1.1 reactivity adjusted map as the base map. Different from previous approaches^22^, the reactivity of single exposure sera was assumed to adopt a cone-like shape, with its apex at the serum coordinate and its height equal to the maximum titer, decreasing at a constant rate of one two-fold per antigenic unit (slope = 1 log2 unit). The assumption of a slope = 1 does not pertain to more cross-reactive sera exposed to multiple variants, hence the serum coordinates and landscape slope for multi-exposure sera, excluded from the initial map, were fitted. Using R’s^41^ (Version 4.2.0) “optim” function with the parameters maxit=500 and the optimization method “L-BFGS-B”, the error between Euclidean map distance and measured titer distance was minimized to obtain x- and y-coordinates for each serum and the landscape’s slope per serum group. P.1.1 measured titers were reduced by one two-fold to account for likely assay reactivity bias. The GMT landscapes show the average of all individual serum landscapes per serum group. For GMT calculation, the reactivity bias of individual sera was accounted for as described in^14^. Landscapes were plotted using the landscapes package (Version 1.1.0)^42^.

### Statistical analysis

Statistical analysis was performed using non-parametric repeated measures ANOVA with Friedman’s test for multiple comparisons. Intervals between the last exposure and blood collection were expressed as mean and interquartile ranges. Statistical analysis were performed using GraphPad Prism (Version 9.4).

### Reporting summary

Further information on research design is available in the Nature Portfolio Reporting Summary linked to this article.

## Supplementary information

Supplementary Materials

Reporting Summary

## Data Availability

Data is publicly available as GitHub repository acorg/roessler_netzl_et_al2022 (https://github.com/acorg/roessler_netzl_et_al2022/tree/v1.0.0)^43^.

## Code availability

The code for the antigenic cartography is publicly available as GitHub repository acorg/roessler_netzl_et_al2022 (https://github.com/acorg/roessler_netzl_et_al2022/tree/v1.0.0)^43^.

## References

[1] Rössler, A., Riepler, L., Bante, D., von Laer, D. & Kimpel, J. SARS-CoV-2 omicron variant neutralization in serum from vaccinated and convalescent persons. *N. Engl. J. Med.*10.1056/NEJMc2119236 (2022). 10.1056/NEJMc2119236 PMC8781314 35021005

[2] Rössler, A., Knabl, L., von Laer, D. & Kimpel, J. Neutralization Profile after Recovery from SARS-CoV-2 Omicron Infection. *N. Engl. J. Med.*10.1056/NEJMc2201607 (2022). 10.1056/NEJMc2201607 PMC9006769 35320661

[3] Carreno, J. M. et al. Activity of convalescent and vaccine serum against SARS-CoV-2 Omicron. *Nature*10.1038/s41586-022-04399-5 (2021). 10.1038/s41586-022-04399-5 35016197

[4] Cele S (2022). Omicron extensively but incompletely escapes Pfizer BNT162b2 neutralization. Nature.

[5] Tarke A (2022). SARS-CoV-2 vaccination induces immunological T cell memory able to cross-recognize variants from Alpha to Omicron. Cell.

[6] Keeton R (2022). T cell responses to SARS-CoV-2 spike cross-recognize Omicron. Nature.

[7] GeurtsvanKessel CH (2022). Divergent SARS-CoV-2 Omicron-reactive T and B cell responses in COVID-19 vaccine recipients. Sci. Immunol..

[8] Wratil PR (2022). Three exposures to the spike protein of SARS-CoV-2 by either infection or vaccination elicit superior neutralizing immunity to all variants of concern. Nat. Med..

[9] Netzl, A. et al. Analysis of SARS-CoV-2 Omicron Neutralization Data up to 2021-12-22. Preprint at bioRxiv 2021.2012.2031.474032 10.1101/2021.12.31.474032 (2022).

[10] Bruel, T. et al. Serum neutralization of SARS-CoV-2 Omicron sublineages BA.1 and BA.2 in patients receiving monoclonal antibodies. *Nat. Med.*10.1038/s41591-022-01792-5 (2022). 10.1038/s41591-022-01792-5 35322239

[11] Bowen JE (2022). Omicron spike function and neutralizing activity elicited by a comprehensive panel of vaccines. Science.

[12] Yu, J. et al. Neutralization of the SARS-CoV-2 Omicron BA.1 and BA.2 Variants. *N. Engl. J. Med.*10.1056/NEJMc2201849 (2022). 10.1056/NEJMc2201849 PMC9006770 35294809

[13] Iketani, S. et al. Antibody evasion properties of SARS-CoV-2 Omicron sublineages. *Nature*10.1038/s41586-022-04594-4 (2022). 10.1038/s41586-022-04594-4 PMC9021018 35240676

[14] Wilks, S. H. et al. Mapping SARS-CoV-2 antigenic relationships and serological responses. Preprint at bioRxiv 2022.2001.2028.477987 10.1101/2022.01.28.477987 (2022). 10.1126/science.adj0070 PMC12145880 37797027

[15] van der Straten K (2022). Antigenic cartography using sera from sequence-confirmed SARS-CoV-2 variants of concern infections reveals antigenic divergence of Omicron. Immunity.

[16] Neerukonda, S. N. et al. SARS-CoV-2 Delta Variant Displays Moderate Resistance to Neutralizing Antibodies and Spike Protein Properties of Higher Soluble ACE2 Sensitivity, Enhanced Cleavage and Fusogenic Activity. *Viruses* 13 10.3390/v13122485 (2021). 10.3390/v13122485 PMC8707919 34960755

[17] Hu, Y.-F. et al. Computation of Antigenicity Predicts SARS-CoV-2 Vaccine Breakthrough Variants. *Front. Immunol.* 13 10.3389/fimmu.2022.861050 (2022). 10.3389/fimmu.2022.861050 PMC8987580 35401572

[18] Mykytyn AZ (2022). Antigenic cartography of SARS-CoV-2 reveals that Omicron BA.1 and BA.2 are antigenically distinct. Sci. Immunol..

[19] Knabl L (2021). High SARS-CoV-2 seroprevalence in children and adults in the Austrian ski resort of Ischgl. Commun. Med (Lond.).

[20] Smith DJ (2004). Mapping the antigenic and genetic evolution of influenza virus. Science.

[21] Bekliz M (2022). Neutralization capacity of antibodies elicited through homologous or heterologous infection or vaccination against SARS-CoV-2 VOCs. Nat. Commun..

[22] Fonville JM (2014). Antibody landscapes after influenza virus infection or vaccination. Science.

[23] Wang, W. et al. Antigenic cartography of well-characterized human sera shows SARS-CoV-2 neutralization differences based on infection and vaccination history. *Cell host & microbe*10.1016/j.chom.2022.10.012 (2022). 10.1016/j.chom.2022.10.012 PMC9584854 36356586

[24] Tuekprakhon A (2022). Antibody escape of SARS-CoV-2 Omicron BA.4 and BA.5 from vaccine and BA.1 serum. Cell.

[25] Liu C (2022). The antibody response to SARS-CoV-2 Beta underscores the antigenic distance to other variants. Cell host microbe.

[26] Cele S (2022). SARS-CoV-2 prolonged infection during advanced HIV disease evolves extensive immune escape. Cell host microbe.

[27] Khan K (2022). Omicron infection enhances Delta antibody immunity in vaccinated persons. Nature.

[28] Seaman, M. S. et al. Vaccine breakthrough infection leads to distinct profiles of neutralizing antibody responses by SARS-CoV-2 variant. *JCI Insight* **7**, 10.1172/jci.insight.159944 (2022). 10.1172/jci.insight.159944 PMC9675445 36214224

[29] Corbett KS (2021). Protection against SARS-CoV-2 Beta variant in mRNA-1273 vaccine-boosted nonhuman primates. Science.

[30] Ebinger JE (2021). Antibody responses to the BNT162b2 mRNA vaccine in individuals previously infected with SARS-CoV-2. Nat. Med.

[31] Lozano-Ojalvo D (2021). Differential effects of the second SARS-CoV-2 mRNA vaccine dose on T cell immunity in naive and COVID-19 recovered individuals. Cell Rep..

[32] Sokal A (2021). mRNA vaccination of naive and COVID-19-recovered individuals elicits potent memory B cells that recognize SARS-CoV-2 variants. Immunity.

[33] Goel, R. R. et al. Distinct antibody and memory B cell responses in SARS-CoV-2 naïve and recovered individuals following mRNA vaccination. *Sci. Immunol.* **6**, 10.1126/sciimmunol.abi6950 (2021). 10.1126/sciimmunol.abi6950 PMC8158969 33858945

[34] Pfizer. Pfizer and BioNTech announce Omicron-adapted COVID-19 vaccine candidates demonstrate high immune response against Omicron, https://www.pfizer.com/news/press-release/press-release-detail/pfizer-and-biontech-announce-omicron-adapted-covid-19 (2022).

[35] Chalkias S (2022). A bivalent omicron-containing booster vaccine against Covid-19. N. Engl. J. Med..

[36] Branche, A. R. et al. SARS-CoV-2 variant vaccine boosters trial: preliminary analyses. Preprint at medRxiv 2022.2007.2012.22277336 10.1101/2022.07.12.22277336 (2022).

[37] Cameroni E (2022). Broadly neutralizing antibodies overcome SARS-CoV-2 Omicron antigenic shift. Nature.

[38] Zhou H, Dcosta BM, Landau NR, Tada T (2022). Resistance of SARS-CoV-2 omicron BA.1 and BA.2 variants to vaccine-elicited sera and therapeutic monoclonal antibodies. Viruses.

[39] Riepler, L. et al. Comparison of Four SARS-CoV-2 Neutralization Assays. Vaccines (Basel) 9 10.3390/vaccines9010013 (2020). 10.3390/vaccines9010013 PMC7824240 33379160

[40] Wilks, S. Racmacs: R Antigenic Cartography Macros, https://acorg.github.io/Racmacs/index.html

[41] R Core Team. *R: A Language and Environment for Statistical Computing*, https://www.R-project.org/ (2021).

[42] Wilks, S. *Antibody landscapes R package*, https://github.com/acorg/ablandscapes

[43] Netzl, A. *acorg/roessler\_netzl\_et\_al2022: Manuscript accepted*, 10.5281/zenodo.7341691 (2022).

