# Supplementary material for: BA.2 and BA.5 omicron differ immunologically from both BA.1 omicron and pre-omicron variants
Source: Nat Commun. 2022 Dec 13;13:7701. doi: 10.1038/s41467-022-35312-3 (PMC9745279; doi:10.1038/s41467-022-35312-3)
Supplement: Supplementary file 1 — Supplementary Materials [file 41467_2022_35312_MOESM1_ESM.pdf]

Supplementary Appendix

Table of Contents

List of Investigators 2
Supplementary Methods 3
Supplementary Figures 8
Supplementary Tables 28
Supplementary References 31

List of Investigators

Annika Rössler[1,*]

Antonia Netzl[2,*]

Ludwig Knabl[3]

Helena Schäfer[1]

Samuel H. Wilks[2]

David Bante[1]

Barbara Falkensammer[1]

Wegene Borena[1]

Dorothee von Laer[1]

Derek Smith[2,#]

Janine Kimpel[1,#]

[1]Institute of Virology, Department of Hygiene, Microbiology and Public Health, Medical University of Innsbruck, Peter-Mayr-Str. 4b, 6020 Innsbruck, Austria

[2]University of Cambridge, Center for Pathogen Evolution, Department of Zoology, Cambridge, UK

[3]Tyrolpath Obrist Brunhuber GmbH, Hauptplatz 4, 6511, Zams, Austria

*Contributed equally

[#]Corresponding authors:

Derek Smith (djs200@cam.ac.uk) and Janine Kimpel (Janine.Kimpel@i-med.ac.at)

Supplementary Methods

Patient Characteristics

Patients were divided into three groups depending on their numbers of exposure to infection/vaccination. Single exposure was defined as unvaccinated with a single infection (n=10 infected during first wave (ancestral virus), n=10 alpha, n=8 beta, n=7 delta, n=18 BA.1 omicron, and n=12 BA.2 omicron. For some of the BA.1 or BA.2 single exposure samples more than one time point was available. For this study, we selected the time point with the peak neutralizing antibody titers against the exposed variant. Two close exposures were defined as two vaccine doses without any history of infection (two homologous doses of mRNA-1273 (n=10), ChAdOx-S1 (n=10) or BNT162b2 (n=11) respectively or heterologous vaccination with ChAdOX-S1 as first and BNT162b2 as second dose (n=10)) and two distant exposures as unvaccinated individuals with two subsequent infections with antigenically distinct variants (n=15 with pre-omicron infection followed by BA.1 re-infection and n=3 with pre-omicron infection followed by BA.2 re-infection). Three or more close exposures were defined as three doses of BNT162b2 vaccination (n=7) and three or more distant exposures as vaccinated individuals with breakthrough infections (n=6) with delta breakthrough after two doses of ChAdOx-S1, n=22 with delta breakthrough after two doses of BNT162b2, n=14 with two or three vaccine doses and BA.1 omicron breakthrough, and n=8 with two or three vaccine doses and BA.2 omicron breakthrough) or vaccinated individuals with a previous pre-omicron breakthrough followed by a BA.1 omicron breakthrough (n=11).

Map construction

Antigenic cartography was first used to study the evolution of influenza A/H3N2[1] and has since been applied to other viruses, including SARS-CoV-2.[2] It uses multi-dimensional scaling[3] to reduce the dimensions of high dimensional titer datasets, which allows the visualization of antigenic relationships, as determined by antibody neutralization assays, in two or three dimensions. Starting from a titer table with *i* rows for *i* antigens and *n* columns for *n* sera, where cell t_{in} denotes the titer of serum *n* against antigen *i*, we create a distance table by $\log_2$ transforming the titers and calculating, for each serum-antigen pair, the difference between the maximum titer in a serum and the titer against the specific antigen:

$$D_{in} = max_i(log_2(t_{in})) - log_2(t_{in}))$$

This distance table gives the target distances for the map, also referred to as table distances. The map distances are optimized such that the sum of squared errors between Euclidean distance *d* between antigens and sera in the reduced dimensions and the target distance *D* is minimized, giving the following stress function:

$$\sum_{i,n} (D_{in} - d_{in})^2$$

As target distances are titer differences on the $\log_2$-scale, one unit map distance corresponds to one two-fold dilution step in the neutralization assay and antigenic map distances reflect neutralization properties of antigen variants. Constructing the map based on fold changes and not on absolute titers allows to control for serum magnitude biases and the magnitude variation within serum groups, which could be due to some sera showing higher reactivity than others. As an example, assuming two sera in the same serum group with highest titers of 1280 and 640, respectively, and variant titers of 320 and 160 gives a fold change of 4 for both sera. To account for variation within the same serum group, each

serum has its own position independent of other sera. This allows sera with high cross-neutralizing responses to be positioned centrally, whereas highly specific sera will be positioned close to their maximum titer variant. A more detailed description of the method can be found in [1,2,4].

For titers below the limit of detection, map distances smaller than the target distance are penalized but greater distances are accepted without a penalty. If a serum or antigen has too many titers below the limit of detection, it cannot be accurately triangulated relative to the other map objects. For the map here, 2 delta, 1 alpha, 4 BA.1 omicron and 3 BA.2 omicron convalescent sera did not have enough detectable titers to be reliably positioned; 2 beta and 6 BA.1 omicron sera were too under constrained in two dimensions and were not positioned at all.

Sera in different map areas increase the resolution of the map (section *Map resolution*, Figure S19*)*.

Map Diagnostics

Map diagnostic tools were used to assess the quality of the antigenic map with P.1.1 reactivity adjusted by -1 on the $\log_2$ scale (Figure 3, Supplementary Figure S2).

Map Dimensionality

First, a dimensionality test was performed with *Racmacs'* [4] *dimensionTestMap* function in dimensions 1 to 5 with a test proportion of 0.1, the minimum column basis set to “none”, the fixed column bases of the current map, and 100 replicates per dimension with 1000 optimizations per replicate. The Root Mean Square Error RMSE between measured and predicted titers was used to judge which dimensionality best represents the antigenic relationships of the titer data. Figure S3 shows that the error between predicted and measured titers is smallest in two dimensions, indicating that the titer data is best represented by a 2D map.

Correlation of fitted and measured titers

Next, the correlation of fitted and measured titers of the map was assessed by converting map distances into $\log_2$ titers. As one antigenic unit in the map corresponds to one two-fold dilution in the neutralization assay, subtracting the Euclidean distance for each serum-antigen pair from the maximum $\log_2$ titer of the specific serum gives $\log_2$ titers.

Doing this for each serum group revealed good correlation of measured and fitted $\log_2$ titers (Figure S4). In the omicron convalescent serum groups, measured titers exceeded fitted titers slightly. A large number of below detectable titers, however, impairs the accurate positioning of a serum and explains the larger difference between measured and fitted titers in the omicron convalescent compared to other serum groups.

Map robustness

Bootstrapping on sera and antigens was performed to determine how robust the map is to measurement noise and the exclusion of samples and antigen variants. *Racmacs'* [4] *bootstrapMap* function with the “noisy” method was used to simulate measurement noise, doing 1000 repeats and 100 optimizations per repeat, bootstrapping both sera and antigens with normally distributed

measurement noise with a standard deviation of 0.7 added to either individual titers, all titers against a specific antigen or both (Figure S5). The same function and optimization routine was used to test the impact of serum and antigen exclusion on the map using the “resample” method and resampling only antigens, sera or resampling both (Figure S6). To test the exclusion of entire serum groups (Figure S7) or antigen variants (Figure S8), maps were constructed excluding the specific sera or variant with 500 optimizations per map and the minimum column basis set to “none”.

Adding noise to antigen reactivity adds noise to all titers measured against the specific antigen, whereas titer noise adds noise to a single measurement. The effects of which are shown in Figure S5. The variants’ positions were very robust to titer noise (Figure S5B). The positions of non-omicron variants varied by one to two antigenic units when adding noise to antigenic reactivity (Figure S5 A, C). Antigen noise resulted in hemisphering of BA.1 omicron, referring to two distinct optima for the variant’s position. The big effect on variant position by some noise can be understood if the added noise moves an originally detectable titer below the detection threshold, or vice-versa. In case of the three omicron sub-lineages, many titers were at the border or below detectable, hence adding artificial measurement noise affected the positioning of these variants more than the other variants.

This can also be seen in Figure S6, where 1000 optimization runs were performed in which both antigens and sera were randomly resampled with replacement (Figure S6A), only antigen variants were resampled (Figure S6B) or individual serum samples were randomly sampled with replacement (Figure S6C). Resampling variants had little effect on non-omicron variant positioning, but two different optima were found for BA.1 and BA.5 omicron (Figure S6B). While serum bootstrapping had little effect on both variants and sera (Figure S6C), variant bootstrapping demonstrated the uncertainty of BA.1 and BA.5 omicron convalescent sera positioning, which had detectable titers against their root variant but very low to non-detectable titers against other variants.

However, the distance of omicron sub-lineages from vaccine sera remained stable.

To investigate which serum groups or variants had a particularly large impact on map topology, maps excluding single serum groups (Figure S7) or antigen variants (Figure S8) were constructed. Excluding BA.1 omicron conv. sera had the largest effect on BA.1 and BA.5 positioning, bringing the two closer to together. Removal of other serum groups or antigen variants had no effect on variant positions (Figure S8).

Map cross-validation

For cross validation, *Racmacs’* [4] *dimensionTestMap* function was employed to optimize maps in 2 dimensions with a test proportion of 0.1. 1000 replicates and 500 optimizations per replicate were performed and the difference between measured and predicted titer calculated on the $\log_2$ scale.

Briefly, 1000 optimizations runs were performed in which only 90% of titers were used for map construction and the remaining 10% predicted by subtracting the Euclidean map distance for each serum-antigen pair from the maximum $\log_2$ titer of the specific serum. The mean difference between measured and predicted $\log_2$ titer was less one two-fold for detectable titers (Figure S9). However, this differed largely across serum groups and antigen variants (Figure S10). The variability of BA.1 and BA.2 omicron conv. sera positions, and BA.1 and BA.2 omicron variant positions seen in the bootstrap map resulted in much lower predicted than measured titers for these variants in the specific serum groups. As before, if detectable titers against these variants were excluded from the map creation, their position became inaccurate and hence sera with only detectable titers against BA.1 or BA.2 omicron, which was true for the majority of BA.1 and BA.2 omicron conv. sera, could not be positioned either.

Individual antibody landscapes

Antibody landscapes [5] were constructed as described in *Materials & Methods.* With the map shown in Figure 3 serving as base plane, antibody levels for each serum were plotted in a third dimension and a continuous surface fitted to the available titer data. The reactivity profile of serum groups exposed to a single variant, such as the convalescent and double vaccinated serum groups (Figure S11 A-J), was assumed to adopt a cone like shape, with the highest point at the specific serum coordinate and a constant decrease across antigenic space of one two-fold per antigenic unit.[2] This assumption does not apply to serum groups exposed to more than one variant, as high titers against both encountered variants are expected. For these groups (Figure S11 K-R), each serum's coordinates were fitted to optimize the serum's position in the map shown in Figure 3 and the surface's slope was fitted per serum group, assuming the same decrease of reactivity for individuals with the same exposure history. The GMT landscapes show the average of all individual antibody landscapes.

The vaccine landscapes (Figure S11 G-J) were the most homogeneous in terms of titer magnitudes within the serum group. Further, the different vaccine regiments elicited landscapes similar to each other. The individual antibody profiles of most convalescent groups were homogenous. With the exception of one delta serum with high P.1.1 titers and two BA.2 conv. sera all had highest titers against their homologous variant. Differing antibody profiles could be due to unknown prior infections. The specific delta sample, however, was a PCR-confirmed first infection and P.1.1 infections were extremely rare in Austria. Assay noise, which is not unusual to be within one-two fold, could explain the higher than homologous titers.

To investigate potential prior infection of the two cross-reactive BA.2 omicron conv. samples, maps were created without these two sera and antibody landscapes fitted to their titers (Figure S12). The two sera did not impact the variant positions in the map (Figure S12 A-C). Their antibody profiles, however, looked more similar to BA.2 omicron breakthrough infections (sample G780) or reinfections (sample G776) than to BA.2 omicron first infections (Figure S12 D). As the map without those two sera did not differ from the map including the sera, the general conclusion of BA.2 omicron being positioned between pre-omicron variants and BA.1 omicron did not change.

The antibody landscapes after delta breakthrough were similar in both magnitude and shape independent of Astra Zeneca or Pfizer vaccination (Figure S11N-O), and were further comparable to omicron breakthrough infections. Triple vaccination landscapes (Figure S11M) had a very similar shape to BNT/BNT + delta (Figure S11O) but GMTs against BA.2 and BA.5 omicron were lower than after breakthrough. Breakthrough landscapes after omicron sub-lineage infection or vaccinated with prior infection and BA.1 omicron breakthrough were almost indistinguishable both in titer magnitudes and landscape slope (Figure S11K-L, P-R). Most multi-exposure landscapes exhibited a broad reactivity profile, suggesting high degrees of cross-reactivity and good protection against variants within the current mapped antigenic space.

The largest variation of individual landscapes was found in the BA.1 omicron reinfection and breakthrough serum groups (Figure S11 P, R). To investigate the impact of number of vaccinations and variant of prior infection, these two groups were split into subgroups and antibody landscapes were constructed for each subgroup (Figure S13). This revealed that the landscape shapes of double and triple vaccinated individuals with BA.1 omicron breakthrough infection were almost identical, and the observed variation was due to generally higher titers after three vaccine doses (Figure S13 A-B). The shapes of reinfection landscapes, however, differed depending on the first encountered variant (Figure S13 C-E). Prior infection with D614G resulted in antibody profiles similar to the vaccinated and infected,

and previous infection with vaccination groups, with reactivity peaking in the area of D614G and slowly decreasing towards the omicron variants. Sera infected with delta followed by BA.1 omicron had lower titers in the upper area of the map (D614G, alpha, beta), with individual landscapes having higher BA.1 omicron titers than delta titers, resulting in a landscape shape almost mirroring the D614G landscape. Only one sample was available with a prior alpha infection, which exhibited very low titers against BA.1 omicron and much less cross-reactivity than the other BA.1 reinfection landscape.

Sample size and map topology

To investigate the impact of sample size per serum group on map topology, we first constructed a map from geometric mean titers (GMTs) for each serum group (Figure S15A). For GMT calculation, titers below the limit of detection (LOD) were set to LOD/2. The map corresponded well with the full map (Figure S15B). BA.1 and BA.5 omicron positions changed the most. The error lines in Figure S15C show that the BA.1 GMT sample is pulled towards the center of the map by its titers against delta, BA.2 omicron and beta. The BA.1 position is determined by its serum as titers against BA.1 in other serum groups are <LOD. The change of BA.5's position can be understood by low titers in the BA.1 GMT sample, pushing it away from the top of the map, and high titers in the BA.2 GMT sample, pulling it towards BA.2. Adding simulated measurement noise to the GMTs illustrates the sensitivity of the BA.5 position and the other optimum found in the full map (Figure S16). Excluding individual GMT sera had no effect on map topology (Figure S17).

We also constructed maps using only 1, 2, or 3 randomly selected sera from each serum group (Figure S18). Depending on the random samples, we found more and less variation from the main map than in the GMT map but generally high correspondence of map topology even with a single sample per serum group. We conclude that the map is very robust to different sample sizes.

Map resolution

Sera distributed across the mapped antigenic space increase the overall map resolution. To illustrate, one dimension would be sufficient to position variants based on fold drops in a single serum group. The linear distance between variants corresponds to the fold drop from highest titer variant to the other variants. Adding a second serum group could break this 1D geometry based on where the new sera are located. Sera with similar titers like the first serum group would not change the 1D configuration substantially, for example vaccine and 1st wave convalescent sera. However, adding a serum group with highest titers against a different variant and different fold drop patterns breaks the 1D geometry. Beta sera, found in the "top" of the map, and delta sera, located in the "bottom" of the map add "up/down" resolution. Variants with high titers in beta convalescents will be located in the upper map area, low titer variants in the lower area, and vice versa in the delta convalescent group.

Without sera in a variant's map area the circumferential resolution of its position relative to the others is very low. Constant force loci (Triangulation blobs) show the area that a variant can occupy without the map stress increasing by more than one unit and thus reflect a 2D conference interval that shows a variant's triangulation. A map without BA.1 and BA.2 omicron convalescent sera illustrates the low triangulation for BA.1 without these serum groups (Figure S19A). Titrations in sera in the variant's map area increase the circumferential resolution of its position (Figure S19D-F).

Supplementary Figures

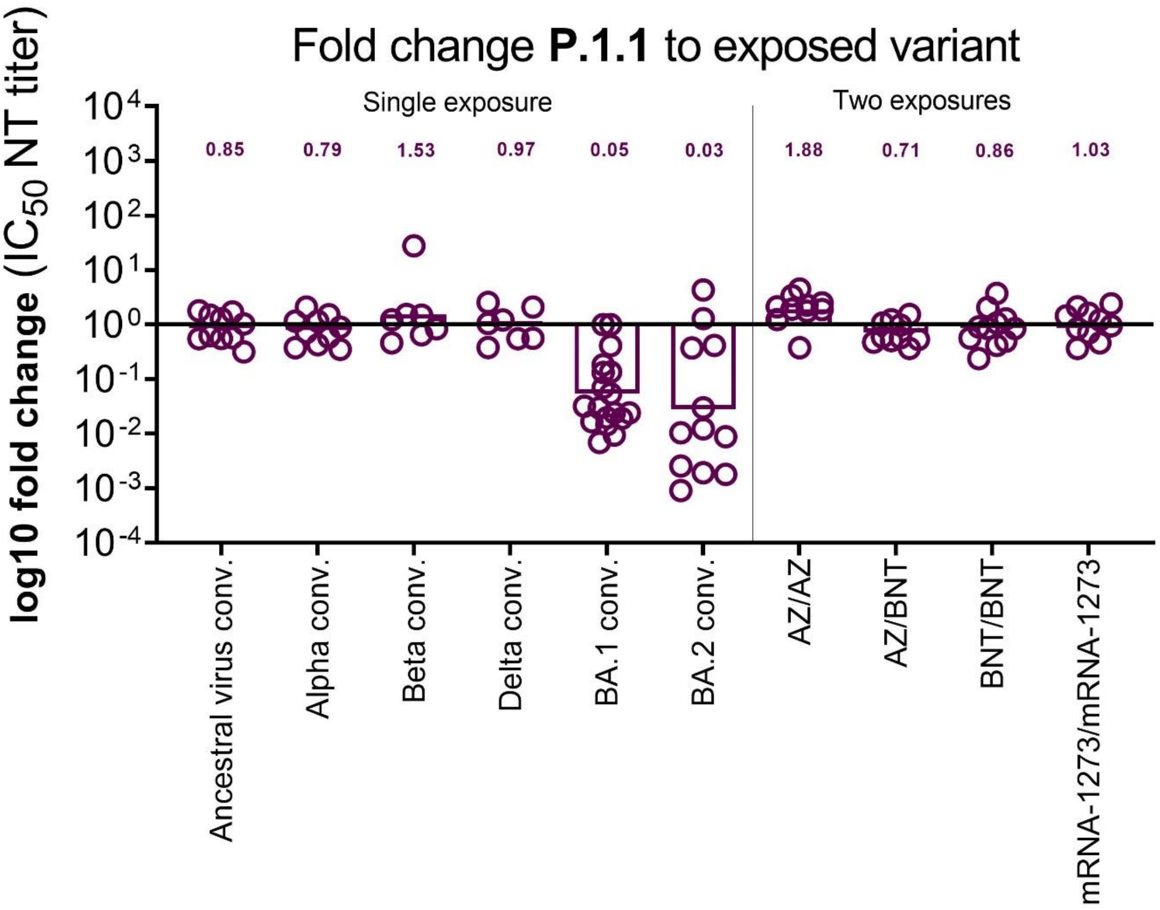

Figure S1. Fold change neutralizing antibody titers gamma versus exposed variant. For each sample (Ancestral virus conv. n=10; Alpha conv. n=10; Beta conv. n=8; Delta conv. n=7; BA.1 conv. n=18; BA.2 conv. n=12; ChAdOx-S1/ChAdOx-S1 n=10; ChAdOx-S1/BNT n=10; BNT/BNT n=11; mRNA-1273/mRNA-1273 n=10), the fold change titer in neutralizing antibodies (IC_{50}) between gamma and the exposed variant was calculated. For ancestral virus convalescent or two dose vaccinated individuals the D614G titers were used as exposed variant. Shown are individual samples and geometric mean change as bars. Numbers indicate the geometric mean change for each group.

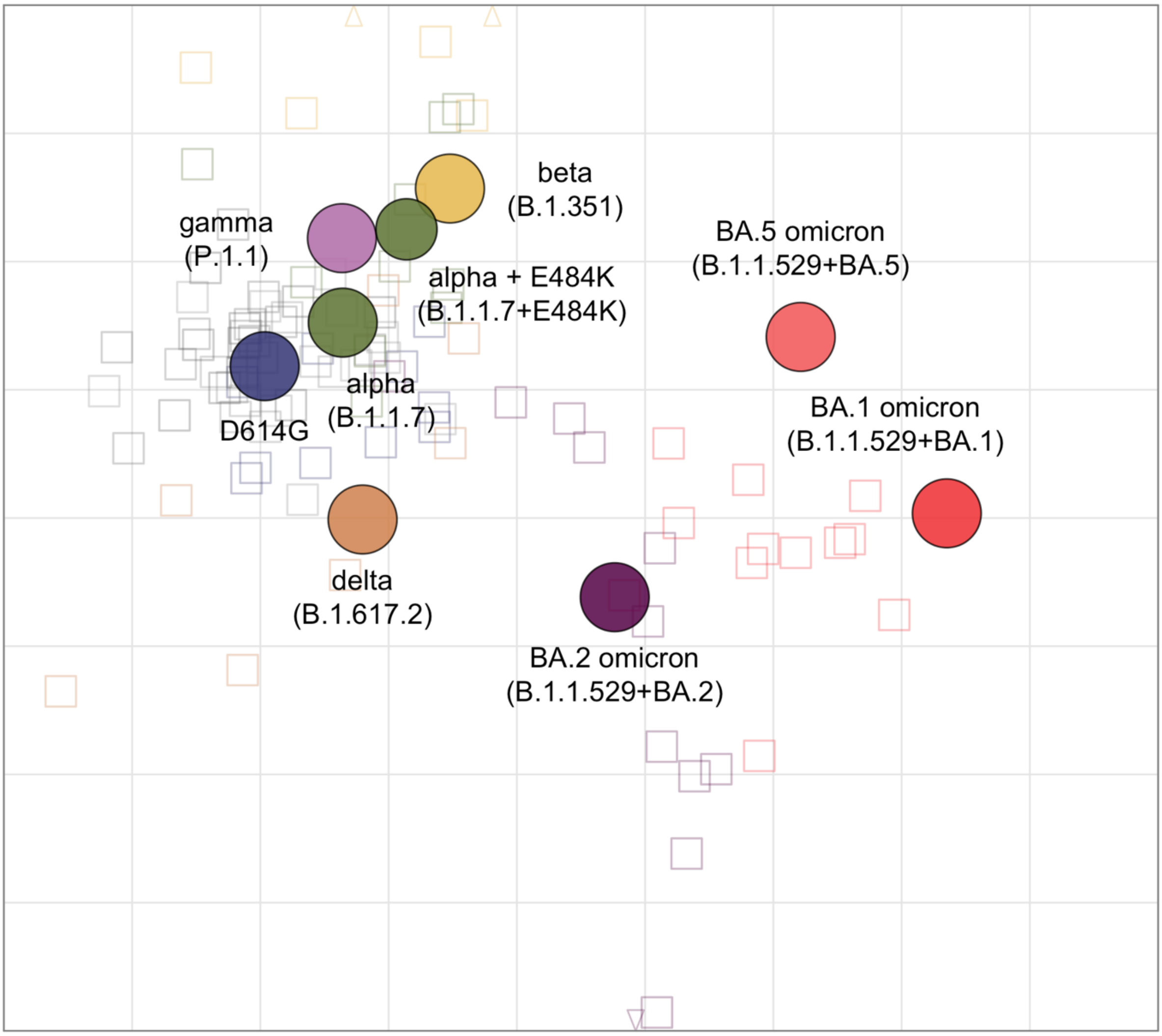

Figure S2. Non-zoomed in version of the antigenic map. Virus variants are shown as colored circles, sera as open squares with the color corresponding to the infecting variant. Triangles point to sera outside the shown map area. Vaccine sera are shown in grey tones (from dark to light: mRNA-1273/mRNA-1273 n=10, BNT162b2/BNT162b2 n=11, ChAdOx-S1/BNT162b2 n=10, ChAdOx-S1/ChAdOx-S1 n=10). The alpha + E484K variant is shown as smaller circle due to its additional substitution compared to the alpha variant. The x- and y-axis represent antigenic distances with one grid square corresponding to one two-fold serum dilution of the neutralization titer. The map orientation within x- and y-axis is free as only relative distances can be inferred. Only single variant exposure sera have been used for construction of the map. N=2 beta convalescent and n=6 BA.1 convalescent samples could not be positioned in the map because of too many <LOD titers. Number of sera from each group positioned on the map : Ancestral virus conv. n=10, Alpha conv. n=10, Beta conv. n=6, Delta conv. n=7, BA.1 omicron conv. n=12, BA.2 omicron conv. n=12. Each of these serum samples has been titrated against D614G, alpha, alpha E484K, beta, gamma, delta, BA.1 omicron, BA.2 omicron, and BA.5 omicron variants.

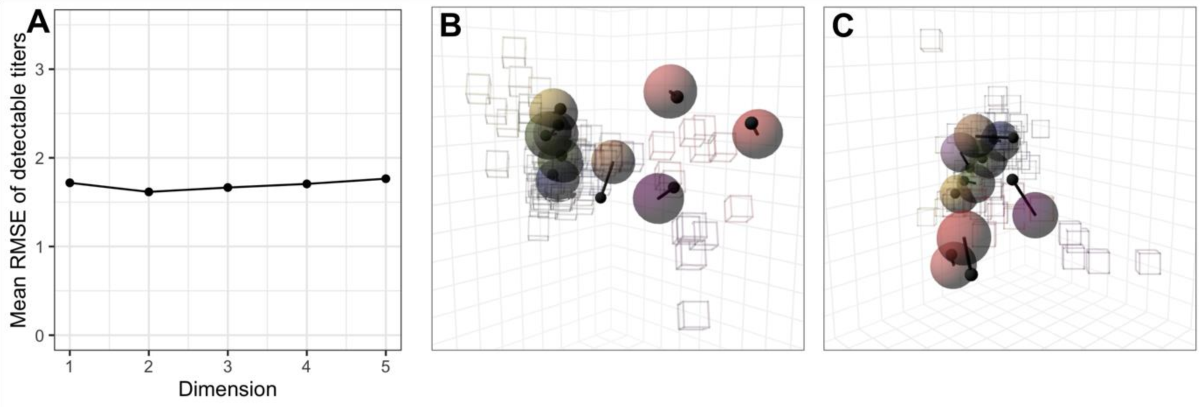

Figure S3. Map dimensionality. A Dimensionality test: RMSE between map and measured titers for detectable titers in 1 to 5 dimensions. Per dimension, 100 map replicates were constructed from 90% of measured titers with 1000 optimizations per replicate. The titers of the remaining 10% were predicted in each run and the RMSE calculated by comparing the predicted to the measured titers on the log_2 scale. **B, C** Side and front view of the map optimized in 3 dimensions with arrows pointing to the variants' position in the 2D map.

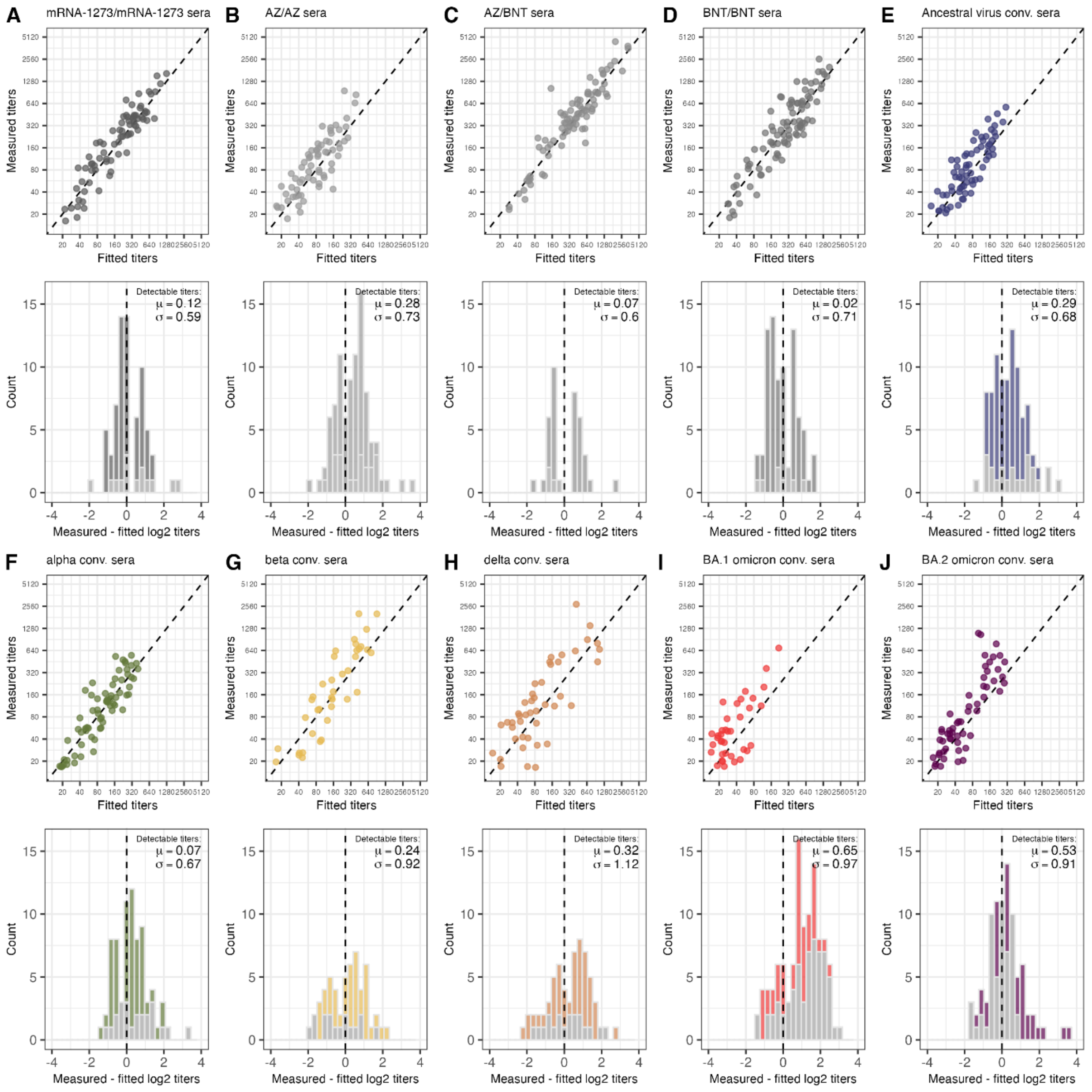

Figure S4. Goodness of map fit per serum group. The top panels show the correlation of detectable measured and fitted titers in the 2D map with P.1.1 reactivity adjustment. Measured P.1.1 titers were reduced by one two-fold to match the reactivity adjustment in the map. Map distances were converted into $\log_2$ titers by subtracting the Euclidean distance for each serum-antigen pair from the maximum $\log_2$ titer of the specific serum. The bottom panels show the residuals of measured against fitted titers on the $\log_2$ scale, light grey marks pairs with the measured titer below the assay detection threshold. The mean and mean-centered standard deviation of differences between fitted and detectable measured titers are given in the legend of each bottom row panel. This was done for the serum groups used to construct the map: **A** mRNA-1273/mRNA-1273, **B** ChAdOx-S1/ChAdOx-S1, **C** ChAdOx-S1/BNT162b2, **D** BNT162b2/BNT162b2, **E** ancestral virus conv., **F** alpha conv., **G** beta conv., **H** delta conv., **I** BA.1 omicron conv., **J** BA.2 omicron conv..

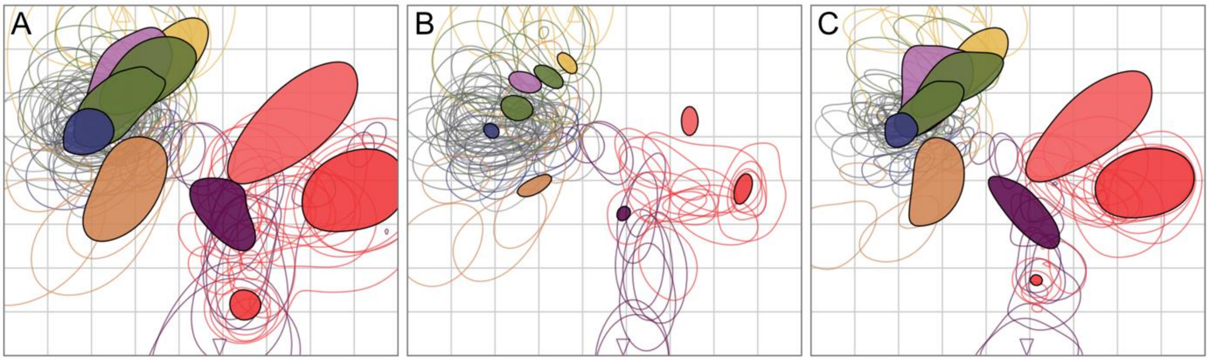

Figure S5. Assessing map robustness to measurement uncertainty by bootstrapping. 1000 bootstrap repeats were performed with 100 optimizations per repeat. Normally distributed measurement noise with a standard deviation of 0.7 was added to **A** titers and antigen reactivity, **B** only titers, and **C** only antigen reactivity. The colored regions mark 68% (one standard deviation) of the positional variation for each variant (filled shapes) and sera (open shapes). The colors correspond to the colors used in Figure 3.

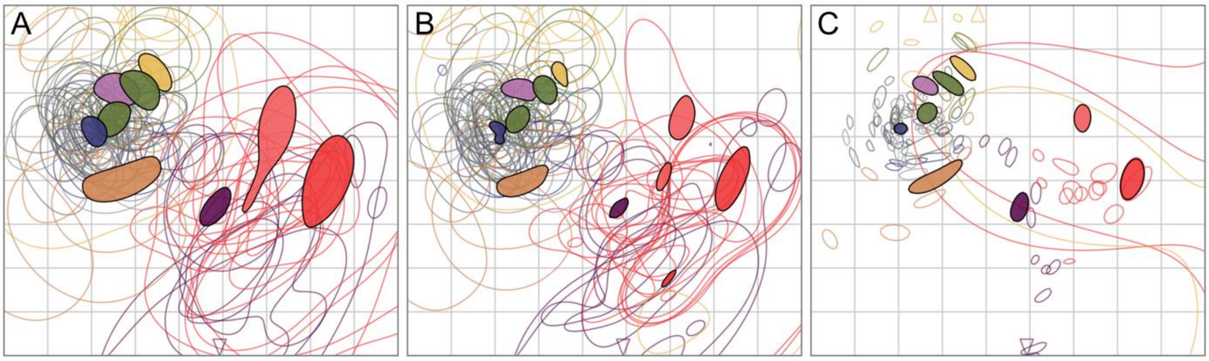

Figure S6. Assessing map robustness to the exclusion of measurements by bootstrapping. 1000 bootstrap repeats were performed with 100 optimizations per repeat. For each repeat, a random subset of titer measurements was taken with replacement. The bootstrapping was performed on **A** variants and sera, **B** only variants, and **C** only sera. The colored regions mark 68% (one standard deviation) of the positional variation for each variant (filled shapes) and sera (open shapes). The colors correspond to the colors used in Figure 3.

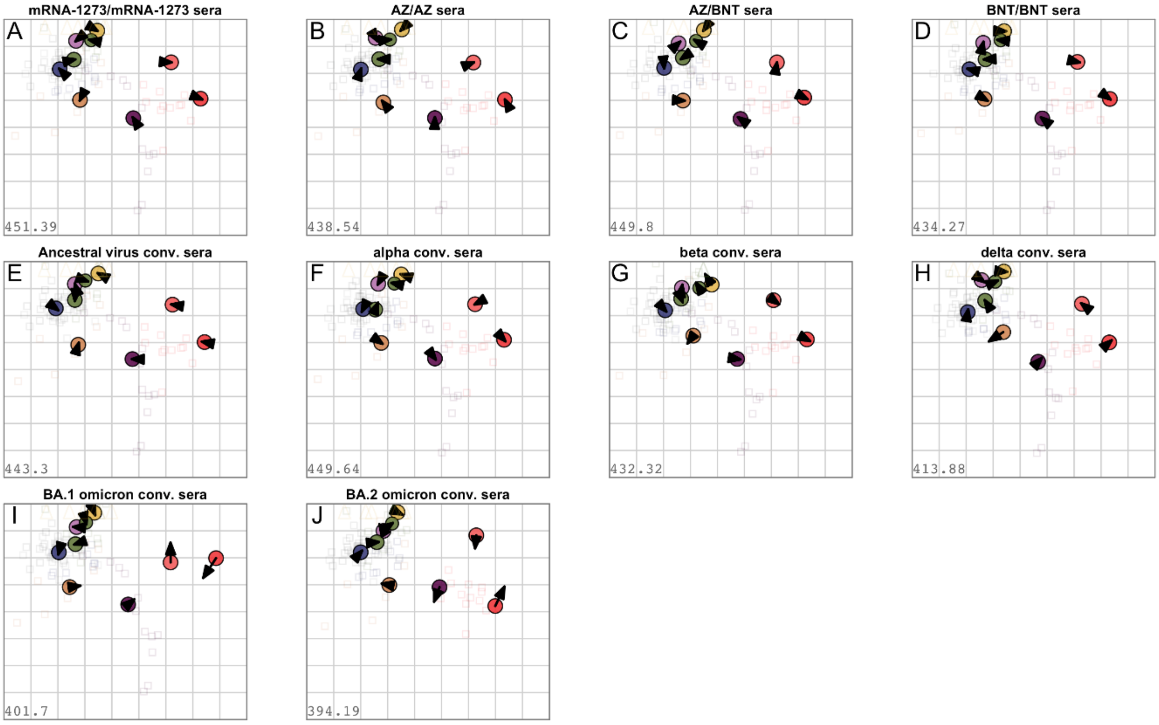

Figure S7. Assessing map robustness to the exclusion of sera. Each serum group was removed and the map reoptimized. Arrows point to the position of each variant in the map shown in Figure 3, for color correspondence refer to this map. A small arrow length indicates similar variant positions and map robustness to the exclusion of the particular serum group. Triangles point to sera positioned outside the plotting area. Maps without **A** mRNA-1273/mRNA-1273, **B** ChAdOx-S1/ChAdOx-S1, **C** ChAdOx-S1/BNT162b2, **D** BNT162b2/BNT162b2, **E** ancestral virus conv., **F** alpha conv., **G** beta conv., **H** delta conv., **I** BA.1 omicron conv., **J** BA.2 omicron conv..

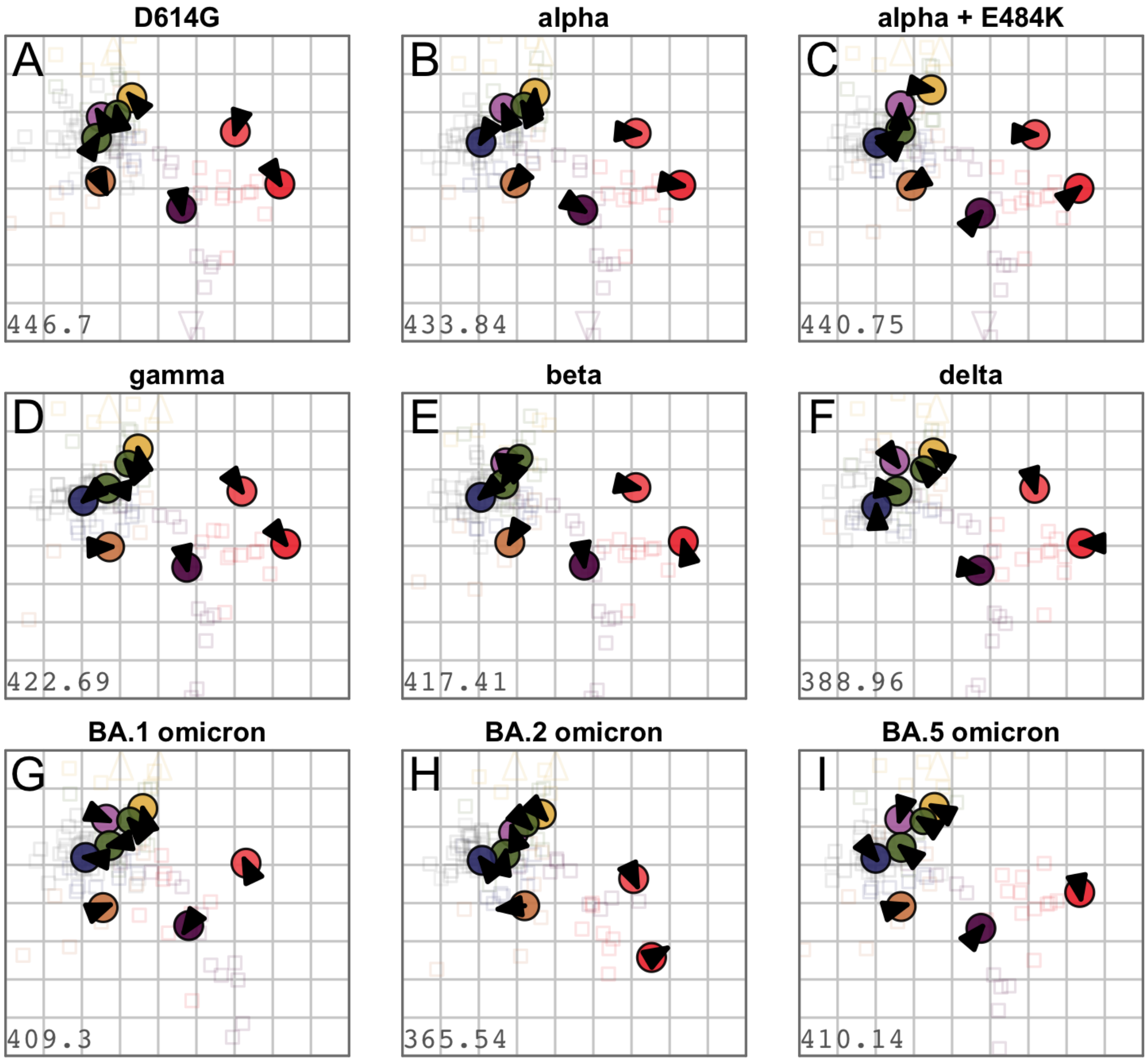

Figure S8. Assessing map robustness to the exclusion of antigen variant. Each antigen variant was removed and the map reoptimized. Arrows point to the position of each variant in the map shown in Figure 3, for color correspondence refer to this map. A small arrow length indicates similar variant positions and robustness to the exclusion of the particular antigen variant. Triangles point to sera positioned outside the plotting area. Maps without **A** D614G, **B** alpha (B.1.1.7), **C** alpha + E484K (B.1.1.7 + E484K), **D** gamma (P.1.1), **E** beta (B.1.351), **F** delta (B.1.617.2), **G** BA.1 omicron (B.1.1.529+BA.1), **H** BA.2 omicron (B.1.1.529+BA.2)**, I** BA.5 omicron (B.1.1.529+BA.5)**.**

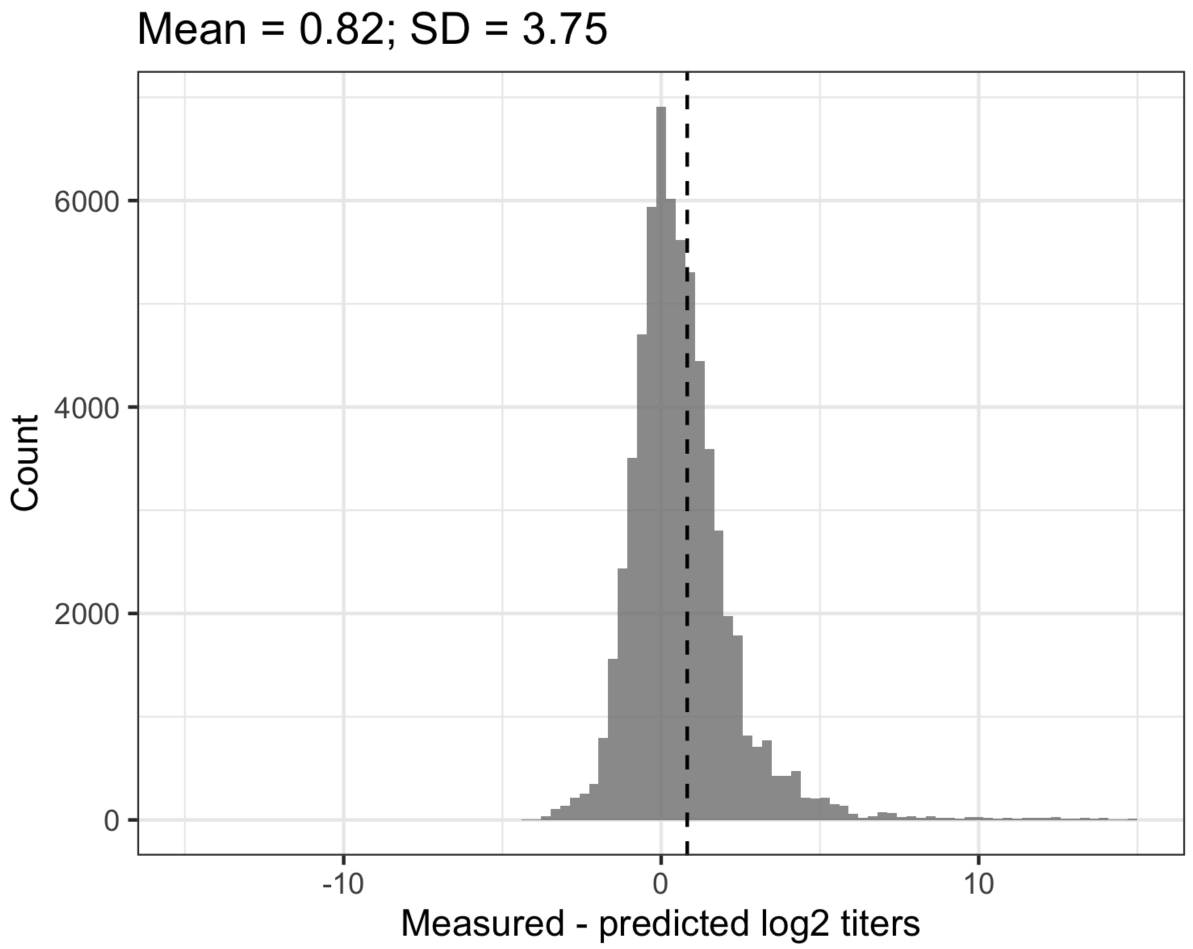

Figure S9. Map cross-validation residual titers. 1000 optimization runs were performed with only 90% of measured titers used for map construction by artificially masking 10% of measurements. The missing log_2 titers were predicted by subtracting the Euclidean map distance for each serum-antigen pair from the maximum log_2 titer of the specific serum. The difference between predicted and detectable measured titers on the log_2 scale was calculated, the mean is indicated by the dashed line. The mean and mean-centered standard deviation are given. The upper x-axis limit has been set to 15 for plotting purposes, very few residuals in the BA.1 and BA.2 omicron convalescent groups were larger than 100, due to inaccurate positioning of sera.

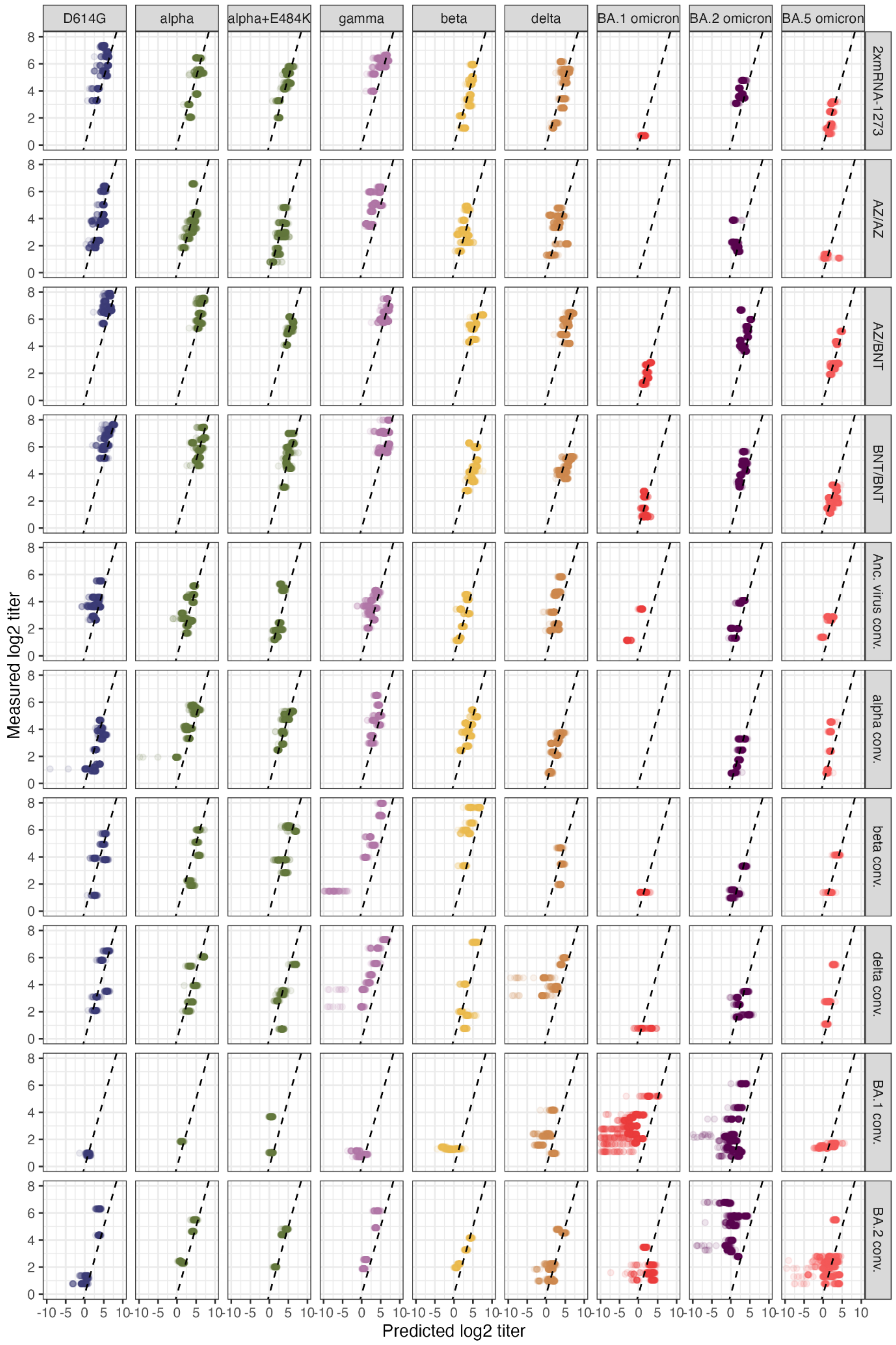

Figure S10: Map cross-validation predicted vs. measured titers. *Figure caption on next page.*

Figure S10. Map cross-validation predicted vs. measured titers. 1000 optimization runs were performed with only 90% of measured titers used for map construction by artificially masking 10% of measurements. The missing log_2 titers were predicted by subtracting the Euclidean map distance for each serum-antigen pair from the maximum log_2 titer of the specific serum. The detectable measured over predicted log_2 titers are shown per serum group and antigen variant. No BA.1 omicron titers were above the detection threshold in the alpha convalescent and AZ/AZ vaccinated serum groups. The lower x-axis limit has been set to -10 for plotting purposes, very few residuals in the BA.1 and BA.2 omicron convalescent groups were larger than 100, due to inaccurate positioning of sera. Many thresholded titers in the original dataset for the omicron variants and in the omicron serum group resulted in inaccurate predictions if the detectable measurements were masked in map making.

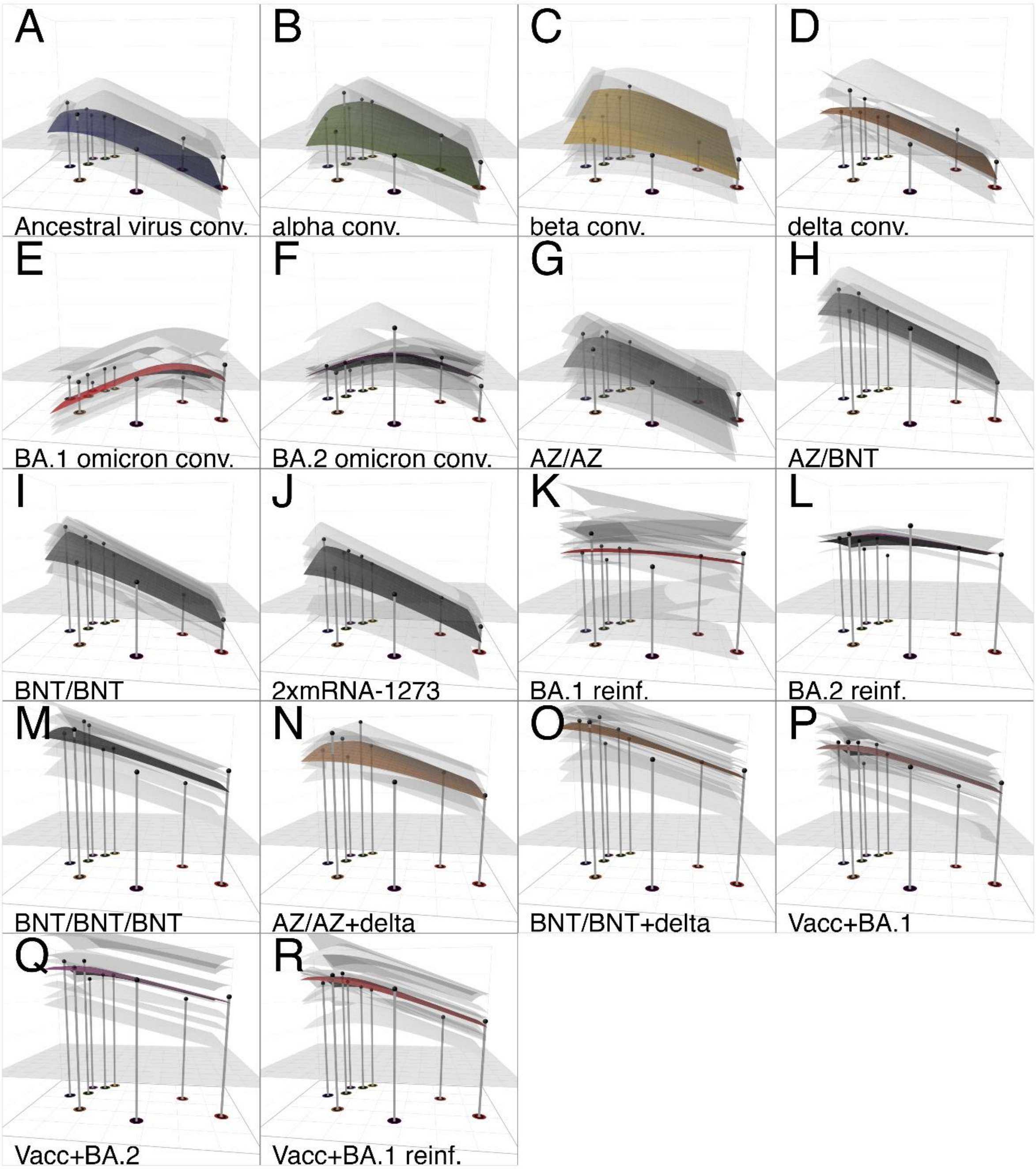

Figure S11. Individual serum antibody landscapes. The colored surfaces represent the GMT antibody landscapes of the different serum groups, the grey transparent surfaces individual serum landscapes. Serum reactivity bias was adjusted for GMT calculation as described in [2]. The map shown in Figure 3 serves as base plane, the z-axis reflects $\log_2$ titers with each two-fold increase marked starting from titer 20. The grey plane at titer 50 serves as reference. The impulses show serum group GMTs for each variant. The landscapes are shown for the following serum groups: **A** Ancestral virus conv. n=10, **B** alpha conv. n=10, **C** beta conv. n=8, **D** delta conv. n=7, **E** BA.1 omicron conv. n=18, **F** BA.2 omicron conv. n=12, **G** ChAdOx-S1/ChAdOx-S1 (AZ/AZ) n=10, **H** ChAdOx-S1/BNT162b2 (AZ/BNT) n=10, **I** BNT162b2/BNT162b2 (BNT/BNT) n=11, **J** mRNA-1273/mRNA-1273 n=10, **K** Pre-Omicron infection + BA.1 omicron reinfection n=15, **L** Pre-Omicron infection + BA.2 omicron reinfection n=3, **M** BNT/BNT/BNT n=7, **N** AZ/AZ + delta breakthrough n=6, **O** BNT/BNT + delta breakthrough n=22, **P** BA.1 omicron breakthrough n=14, **Q** BA.2 omicron breakthrough n=8, **R** Vaccinated, previous non-omicron infection and BA.1 omicron infection n=11. Serum coordinates and landscapes for **K-R** were fitted as described in *Materials & Methods.*

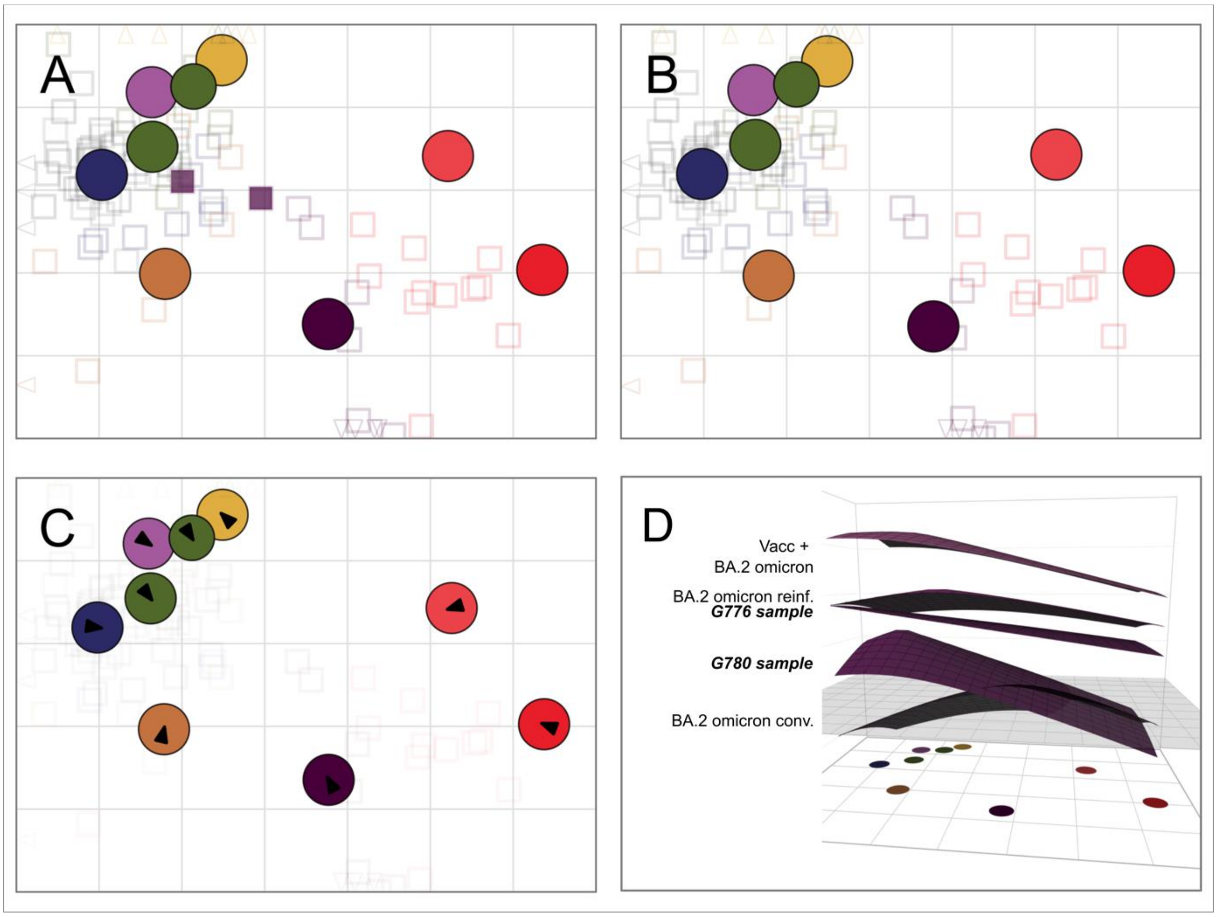

Figure S12. Testing effect of cross-reactive BA.2 omicron convalescent sera on map and landscape. **A** Map in Figure 3 shown with two cross-reactive BA.2 convalescent sera (G776, G780) highlighted in dark purple. **B** A map was constructed excluding these two sera to test their effect on variant positioning. **C** Map in **B** with arrows pointing to the variant's position in map **A.** The small arrows indicate that the two cross-reactive samples do not impact BA.2 positioning. **D** GMT Antibody landscapes of serum groups which encountered BA.2 omicron (Vacc + BA.2 omicron breakthroughs, BA.2 omicron reinfected, BA.2 convalescents, (Fig. S11 O, P, F)) and individual landscapes of the two BA.2 convalescent samples with high titers against non-omicron variants (G776, G780). The slope of the BA.2 conv. landscape, excluding the two cross-reactive samples, was set to 1, all other slopes and serum coordinates were fitted as described in *Material & Methods.* The map shown in panel **B** serves as base plane, the z-axis reflects $\log_2$ titers with each two-fold increase marked starting from titer 20. The grey plane at titer 50 serves as reference.

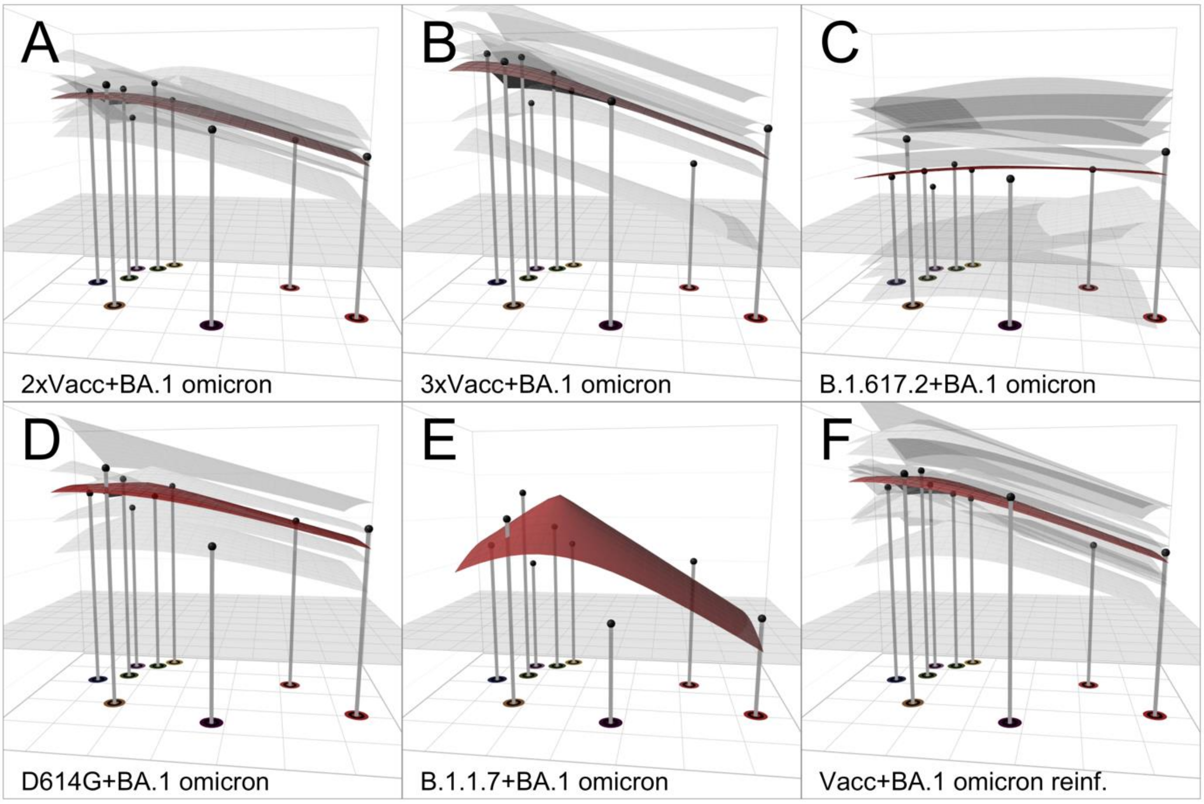

Figure S13. Variability of BA.1 omicron multi-exposure landscapes. Individual (transparent) and GMT (solid) antibody landscapes for the Vacc + BA.1 omicron (**A-B)**, BA.1 omicron reinf. (**C-E)** and Vacc + BA.1 omicron reinf. (**D**) after subsetting by number of vaccinations and prior infection (Fig. S11 M, O). The map in Figure 3 serves as base plane, the serum coordinates per serum and landscape slopes per additional serum group were fitted as described in *Materials & Methods.* Serum reactivity bias was adjusted for GMT calculation as described in [2]. The z-axis reflects $\log_2$ titers with each two-fold increase marked starting from titer 20. The grey plane at titer 50 serves as reference.

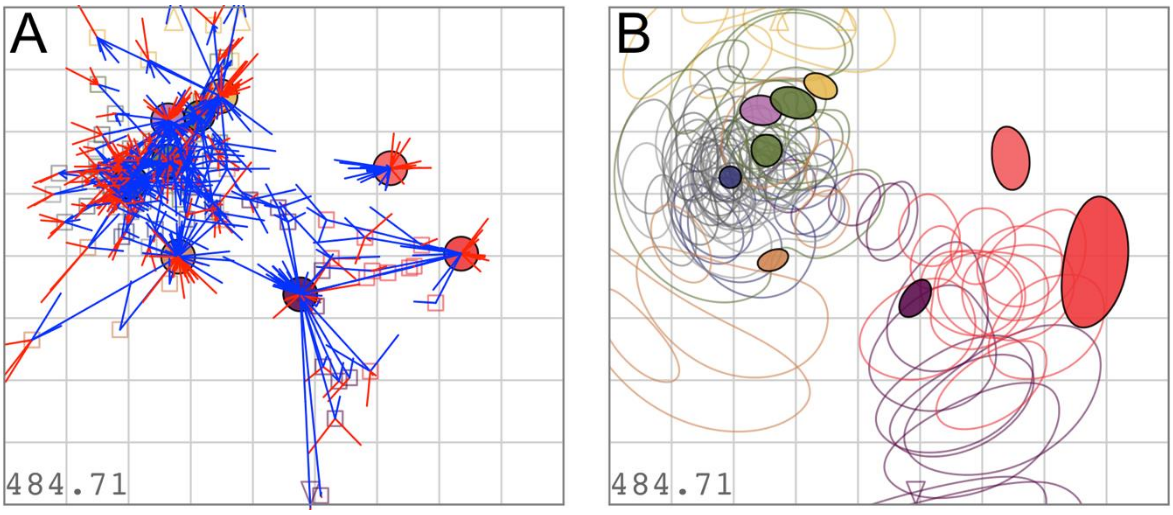

Figure S14. Titer error lines and map triangulation. Samples that could not be positioned due to too many titers <LOD were excluded from this map diagnostics step (n=8). **A** Error lines for each serum and antigen are shown in blue in case of larger map distance than target distance and red in case of smaller map distance than target distance. The length of each error bar indicates the magnitude of mismatch. Blue error lines point towards the variant-serum pair that has a smaller target distance, red error lines point away from the variant-serum pair. **B** Constant force loci (Triangulation blobs) show the area for each serum and variant in which the item can move without increasing map stress by more than one unit. Filled shapes show variant Triangulation blobs, open shapes sera. Colors correspond to the map shown in Figure 3.

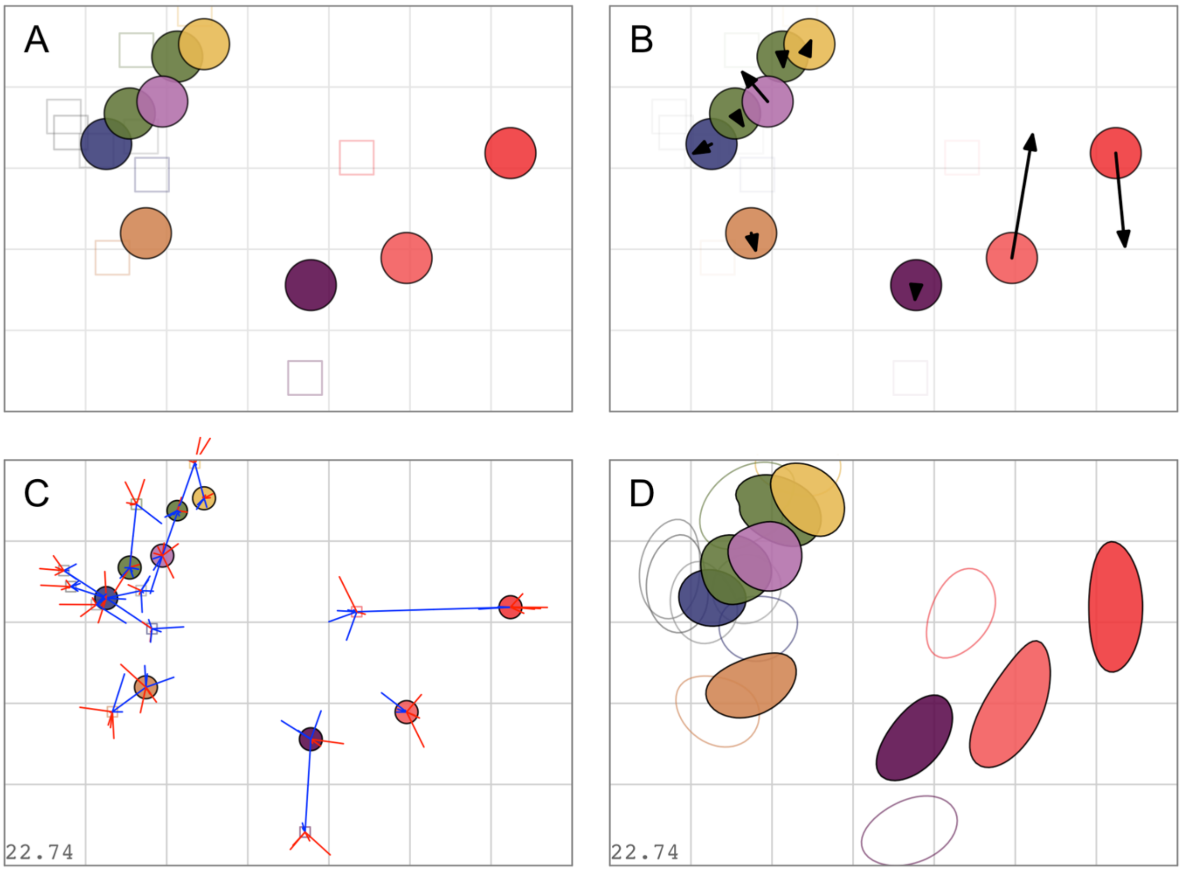

Figure S15. Map from geometric mean titers GMT. A GMTs of each serum groups were used for map construction. The colors correspond to the colors used in Figure 3. **B** Map in A with arrows pointing towards the variants' positions in Figure 3. **C** Error lines connecting GMT sample and variant. Blue lines indicate a larger map than target distance, red lines indicate a smaller map than target distance. **D** Constant force foci (Triangulation blobs) of variants and GMT samples. The marked area corresponds to the area an item can occupy without increasing the map stress by more than 1 unit. Filled shapes show variant Triangulation blobs, open shapes sera.

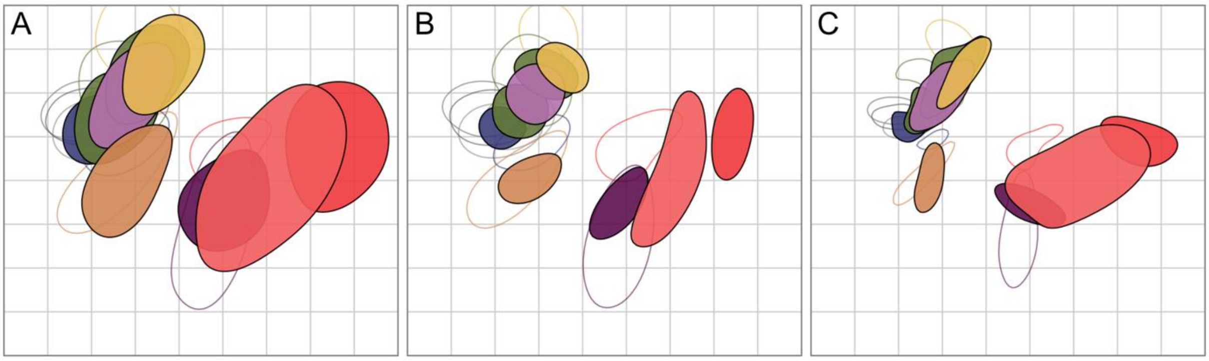

Figure S16. Assessing GMT map robustness to measurement uncertainty by bootstrapping. 1000 bootstrap repeats were performed with 100 optimizations per repeat. Normally distributed measurement noise with a standard deviation of 0.7 was added to **A** titers and antigen reactivity, **B** only titers, and **C** only antigen reactivity. The colored regions mark 68% (one standard deviation) of the positional variation for each variant (filled shapes) and sera (open shapes). The colors correspond to the colors used in Figure 3.

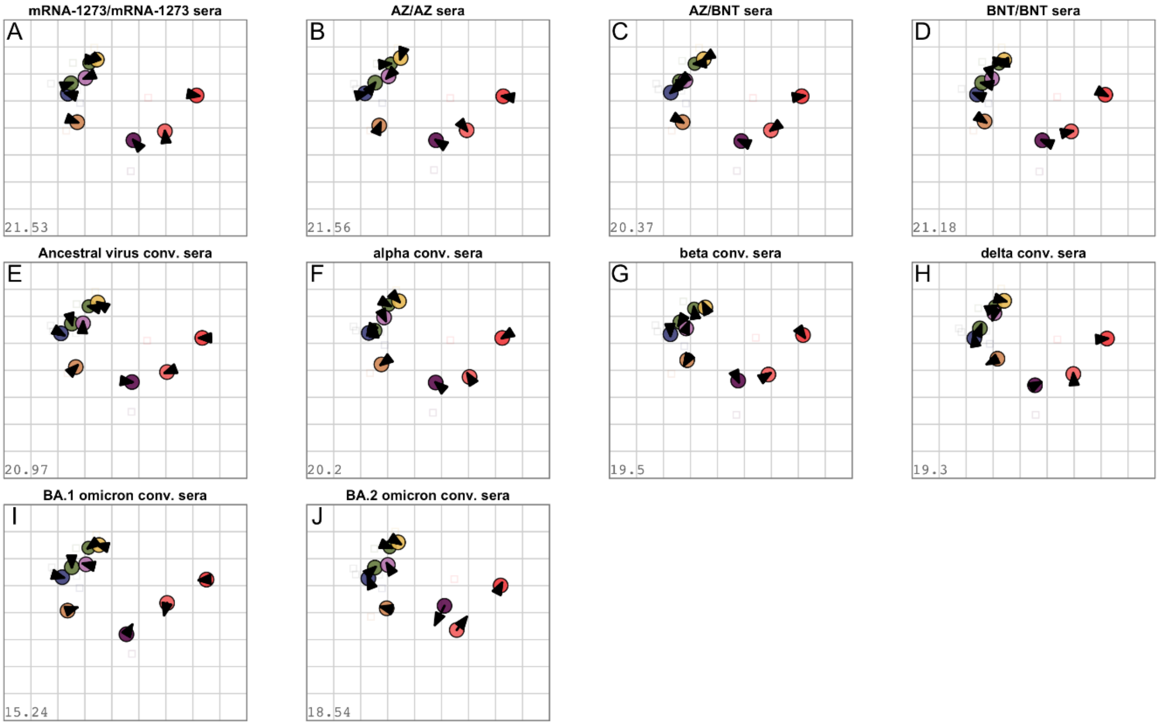

Figure S17. Assessing GMT map robustness to the exclusion of sera. Each serum group was removed and the map re-optimized. Arrows point to the position of each variant in the map shown in Figure 3, for color correspondence refer to this map. A small arrow length indicates similar variant positions and map robustness to the exclusion of the particular serum group. Maps without **A** mRNA-1273/mRNA-1273, **B** ChAdOx-S1/ChAdOx-S1, **C** ChAdOx-S1/BNT162b2, **D** BNT162b2/BNT162b2, **E** ancestral virus conv., **F** alpha conv., **G** beta conv., **H** delta conv., **I** BA.1 omicron conv., **J** BA.2 omicron conv..

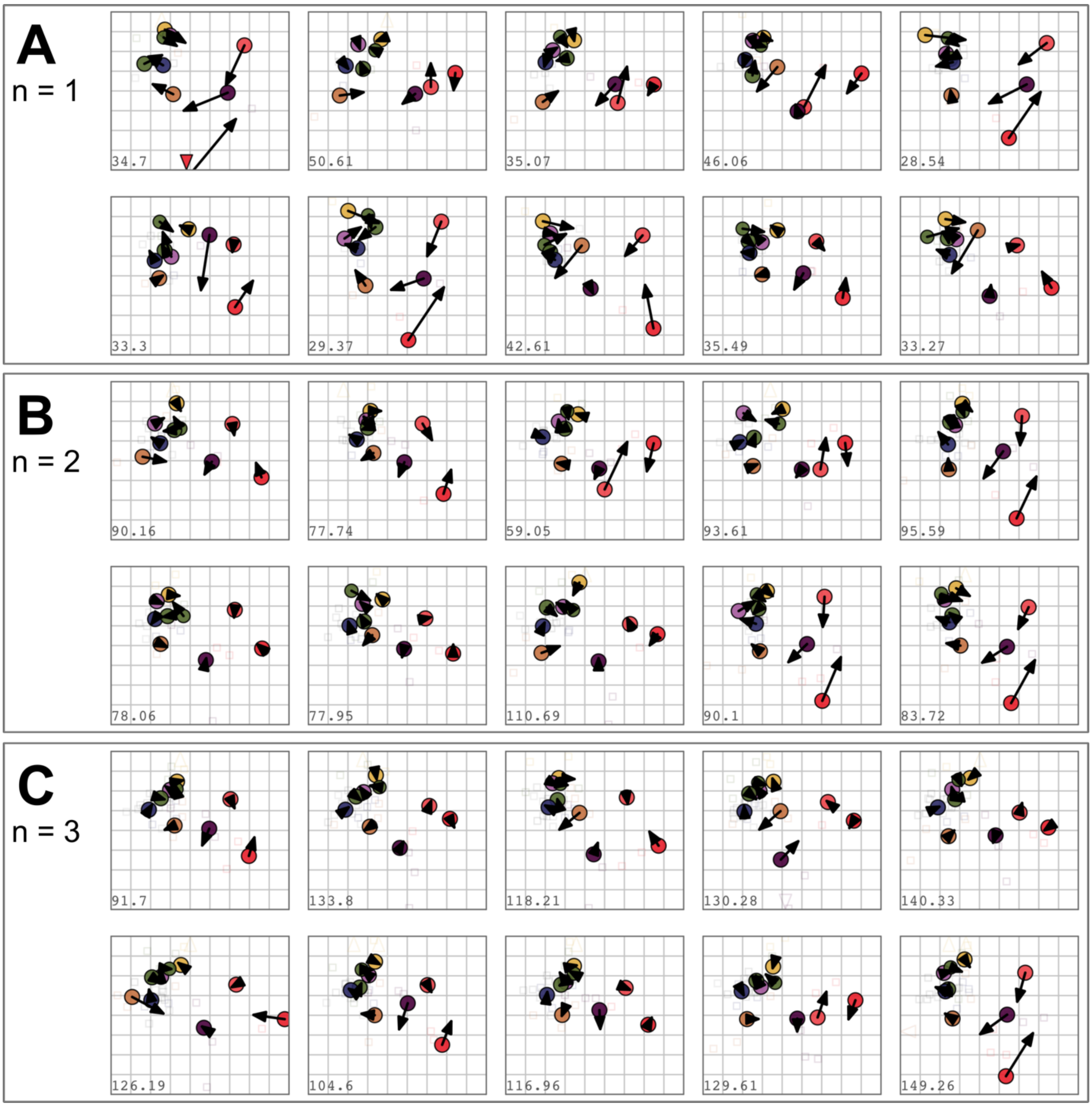

Figure S18. Assessing map robustness to sample size per serum group. Ten Maps were created with a randomly drawn subset of samples with different sample sizes. 1500 optimizations were performed per map with a dilution step size of 0 and the minimum column basis set to “none”. **A** n=1, **B** n=2, **C** n=3 samples were randomly drawn per serum group.

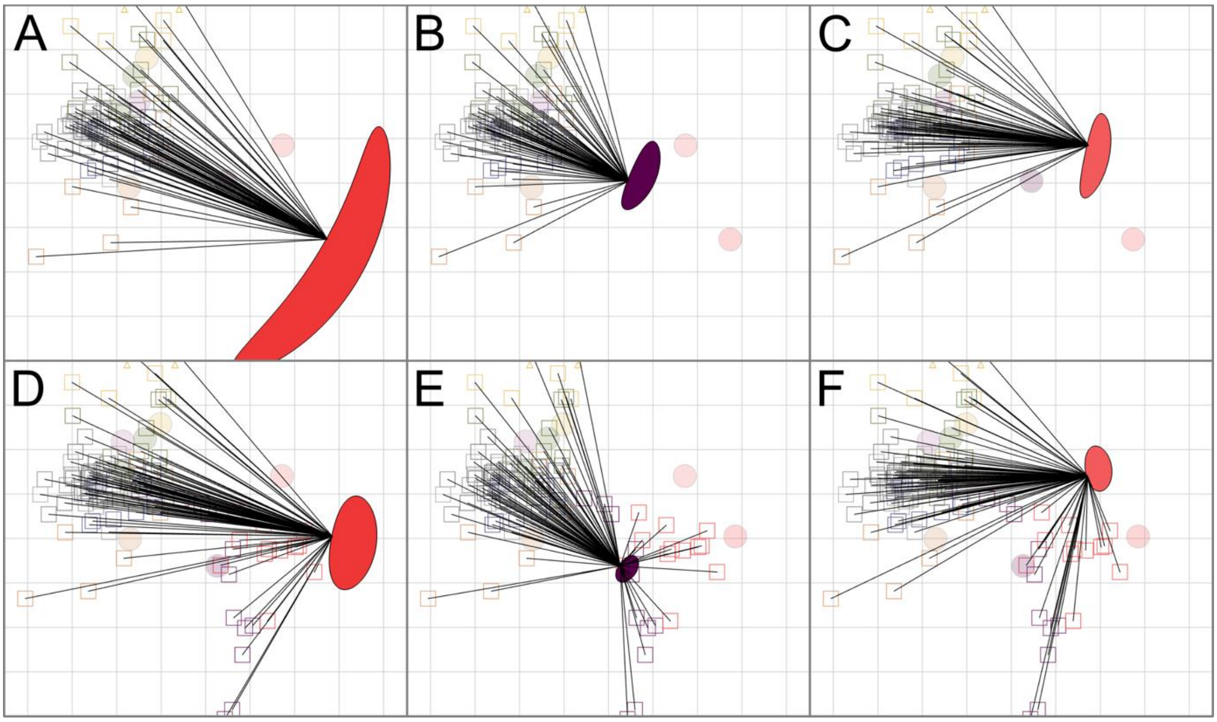

Figure S19. Primary omicron infections increase circumferential resolution. Constant force loci (Triangulation blobs) of the three omicron sub-lineages (left column: BA.1 omicron (**A, D**), middle column BA.2 omicron (**B, E**), right column: BA.5 omicron (**C, F**)) with connection lines of **A-C** a map excluding BA.1 and BA.2 omicron convalescent sera and **D-F** the map shown in Figure 2. The colored area shows the two-dimensional space a variant can occupy without the overall map stress increasing more than one unit. The dark lines connect antigen variants and the sera in which they have been titrated. The colors correspond to the colors used in the main map (Figure 3, S2).

Supplementary Tables

Table S1. Patient Characteristics

Study cohort	number of participants	vaccination (+/-)	Variant of infection		Mean age years ± SD	% female (N)	Blood collection Mean weeks (IQR)*
			first	second			
**Single exposure**							
Ancestral virus convalescent	10	-	ancestral	-	51.6 ± 11.8	60.0 (6)	2 - 6
Alpha convalescent	10	-	alpha	-	49.1 ± 14.3	80.0 (8)	22 (21 - 25)
Beta convalescent	8	-	beta	-	79.8 ± 9.5	50.0 (4)	1 - 4
Delta convalescent	7	-	delta	-	27.3 ± 10.3	42.9 (3)	10 (4 - 18)
BA.1 convalescent	18	-	BA.1 omicron	-	47.0 ± 17.2	61.1 (11)	2.4 (1.7 - 2.3)
BA.2 convalescent	12	-	BA.2 omicron	-	35.8 ± 14.9	50.0 (6)	2.3 (1.9 - 2.4)
**Two exposures**							
AZ/AZ	10	+	-	-	37.3 ± 9.3	80.0 (8)	4.3 (4.3 - 4.3)
AZ/BNT	10	+	-	-	32.8 ± 4.1	70.0 (7)	4.3 (4.3 - 4.3)
BNT/BNT	11	+	-	-	36.1 ± 10.6	63.6 (7)	4.3 (4.3 - 4.3)
mRNA-1273/ mRNA-1273	10	+	-	-	35.4 ± 9.4	60.0 (6)	20 (18 - 23)
BA.1 reinfected	15	-	ancestral (n=5) alpha (n=1) delta (n=9)	BA.1 omicron	35.0 ± 16.9	72.7 (8)	1.8 (1.4 - 2.1)
BA.2 reinfected	3	-	ancestral (n=1) delta (n=2)	BA.2 omicron	34.7 ± 6.2	66.7 (2)	2.8 (2.3 - 3.3)
**≥ Three exposures**							
AZ/AZ + delta	6	+	delta	-	30.3 ± 4.6	100.0 (6)	3 - 12
BNT/BNT + delta	22	+	delta	-	50.7 ± 14.4	59.1 (13)	2 - 4
BNT/BNT/BNT	7	+	-	-	38.7 ± 8.7	71.4 (5)	4.3 (4.3 - 4.3)
Vacc + BA.1	14	+	omicron (BA.1)	-	36.5 ± 16.8	57.1 (8)	1.9 (1.3 - 2.6)
Vacc + BA.2	8	+	omicron (BA.2)	-	32.8 ± 4.1	62.5 (5)	2 - 5
Vacc + BA.1 reinfected	11	+	ancestral (n=4) alpha (n=1) delta (n=6)	BA.1 omicron	36.4 ± 16.8	54.5 (6)	2.1 (1.7 - 2.3)

* Intervals between last exposure and blood collection for the different groups were expressed as mean weeks and interquartile range (IQR) when exact intervals were known. For groups where this was not the case time periods are given in weeks, i.e. for ancestral virus convalescent, beta convalescent, AZ/AZ vaccinated + delta breakthrough, BNT/BNT + delta breakthrough, vaccinated + BA.2 breakthrough.

Table S2. Statistics for neutralizing antibody titers against indicated variant compared to BA.2 omicron.

	Comparison of BA.2 titers against[$]							
**Study cohort**	**D614G**	**Alpha**	**Alpha + E484K**	**Beta**	**Gamma**	**Delta**	**BA.1 Omicron**	**BA.5 Omicron**
**Single exposure**								
BA.2 convalescent (n=12)	0.0486	0.0070	0.0024	0.0002	0.0081	0.0218	0.0486	>0.9999
Ancestral virus convalescent (n=10)	0.0038	0.0253	>0.9999	>0.9999	0.0038	0.0101	>0.9999	>0.9999
**Two exposures**								
AZ/AZ + delta (n=6)	>0.9999	>0.9999	0.0009	>0.9999	>0.9999	>0.9999	>0.9999	>0.9999
BNT/BNT + delta (n=22)	<0.0001	<0.0001	0.0002	>0.9999	<0.0001	0.0002	>0.9999	>0.9999

[$] using non-parametric repeated measures one-way ANOVA with Friedman`s test for multiple comparisons

Table S3. Statistics for neutralizing antibody titers against indicated variant compared to BA.5 omicron.

Study cohort	Comparison of BA.5 titers against[$]							
	**D614G**	**Alpha**	**Alpha + E484K**	**Beta**	**Gamma**	**Delta**	**BA.1 Omicron**	**BA.2 Omicron**
**Single exposure**								
BA.2 convalescent (n=12)	>0.9999	>0.9999	>0.9999	0.0166	>0.9999	>0.9999	>0.9999	>0.9999
Ancestral virus convalescent (n=10)	0.0009	0.0073	>0.9999	>0.9999	0.0009	0.0027	>0.9999	>0.9999
**Two exposures**								
AZ/AZ + delta (n=6)	>0.9999	>0.9999	0.0022	>0.9999	>0.9999	>0.9999	>0.9999	>0.9999
BNT/BNT + delta (n=22)	<0.0001	<0.0001	0.0001	0.0419	<0.0001	0.0001	>0.9999	>0.9999

[$] using non-parametric repeated measures one-way ANOVA with Friedman`s test for multiple comparisons

Supplementary References

1. Smith DJ, Lapedes AS, de Jong JC, et al. Mapping the antigenic and genetic evolution of influenza virus. Science 2004;305(5682):371-6. (In eng). DOI: 10.1126/science.1097211.
2. Wilks SH, Mühlemann B, Shen X, et al. Mapping SARS-CoV-2 antigenic relationships and serological responses. bioRxiv 2022:2022.01.28.477987. DOI: 10.1101/2022.01.28.477987.
3. Kruskal J, Wish M. Multidimensional Scaling. Thousand Oaks, California1978.
4. Wilks S. Racmacs: R Antigenic Cartography Macros. (https://acorg.github.io/Racmacs/index.html).
5. Fonville JM, Wilks SH, James SL, et al. Antibody landscapes after influenza virus infection or vaccination. Science 2014;346(6212):996-1000. DOI: doi:10.1126/science.1256427.
