# Supplementary material for: BA.2 and BA.5 omicron differ immunologically from both BA.1 omicron and pre-omicron variants
Source: Nat Commun. 2022 Dec 13;13:7701. doi: 10.1038/s41467-022-35312-3 (PMC9745279; doi:10.1038/s41467-022-35312-3)
Supplement: Supplementary file 2 — Reporting Summary [file 41467_2022_35312_MOESM2_ESM.pdf]

nature portfolio

Corresponding author(s): Derek Smith & Janine Kimpel

Last updated by author(s): Nov 20, 2022

Reporting Summary

Nature Portfolio wishes to improve the reproducibility of the work that we publish. This form provides structure for consistency and transparency in reporting. For further information on Nature Portfolio policies, see our Editorial Policies and the Editorial Policy Checklist.

Statistics

For all statistical analyses, confirm that the following items are present in the figure legend, table legend, main text, or Methods section.

n/a	Confirmed	
☐	☒	The exact sample size (*n*) for each experimental group/condition, given as a discrete number and unit of measurement
☐	☒	A statement on whether measurements were taken from distinct samples or whether the same sample was measured repeatedly
☐	☒	The statistical test(s) used AND whether they are one- or two-sided<br>*Only common tests should be described solely by name; describe more complex techniques in the Methods section.*
☐	☒	A description of all covariates tested
☐	☒	A description of any assumptions or corrections, such as tests of normality and adjustment for multiple comparisons
☐	☒	A full description of the statistical parameters including central tendency (e.g. means) or other basic estimates (e.g. regression coefficient) AND variation (e.g. standard deviation) or associated estimates of uncertainty (e.g. confidence intervals)
☒	☐	For null hypothesis testing, the test statistic (e.g. *F*, *t*, *r*) with confidence intervals, effect sizes, degrees of freedom and *P* value noted<br>*Give P values as exact values whenever suitable.*
☒	☐	For Bayesian analysis, information on the choice of priors and Markov chain Monte Carlo settings
☒	☐	For hierarchical and complex designs, identification of the appropriate level for tests and full reporting of outcomes
☒	☐	Estimates of effect sizes (e.g. Cohen's *d*, Pearson's *r*), indicating how they were calculated

Our web collection on statistics for biologists contains articles on many of the points above.

Software and code

Policy information about availability of computer code

Data collection	Calculation of 50% neutralizing antibody titers and statistical significance was performed in Graphpad prism version 9.4 as described in the Methods and Supplementary Methods section. The code for antigenic maps and antibody landscapes is available in the GitHub repository roessler_netzl_et_al2022 (https://github.com/acorg/roessler_netzl_et_al2022/tree/v1.0.0). Antigenic maps and antibody landscapes were made in R version 4.2.0 using the Racmacs (version 1.1.35) and ablandscapes (version 1.1.0) packages. Both packages are free and open source available on GitHub and linked in the manuscript's GitHub repository.
Data analysis	Calculation of 50% neutralizing antibody titers and statistical significance was performed in Graphpad prism version 9.4 as described in the Methods and Supplementary Methods section. The code for antigenic maps and antibody landscapes is available in the GitHub repository roessler_netzl_et_al2022 (https://github.com/acorg/roessler_netzl_et_al2022/tree/v1.0.0). Antigenic maps and antibody landscapes were made in R version 4.2.0 using the Racmacs (version 1.1.35) and ablandscapes (Version 1.1.0) packages. Both packages are free and open source available on GitHub and linked in the manuscript's GitHub repository. A link to the GitHub repository is provided in the Code Availability statement of the main manuscript.

For manuscripts utilizing custom algorithms or software that are central to the research but not yet described in published literature, software must be made available to editors and reviewers. We strongly encourage code deposition in a community repository (e.g. GitHub). See the Nature Portfolio guidelines for submitting code & software for further information.

Data

Policy information about availability of data

All manuscripts must include a data availability statement. This statement should provide the following information, where applicable:

- Accession codes, unique identifiers, or web links for publicly available datasets
- A description of any restrictions on data availability
- For clinical datasets or third party data, please ensure that the statement adheres to our policy

Data is publicly available as GitHub repository acorg/roessler_netzl_et_al2022 (https://github.com/acorg/roessler_netzl_et_al2022/tree/v1.0.0).

Human research participants

Policy information about studies involving human research participants and Sex and Gender in Research.

Reporting on sex and gender	Number and percentage of female participants (self-reported) for each of the groups analyzed are given in Supplementary Table 1. However, no information on individual participant level is given. Differences in neutralizing antibody titers between male and female participants were not analyzed due to small group sizes. Additionally, a number of previous studies report no differences in antibody titers between male and female study participants.
Population characteristics	Participants with different numbers of exposure to SARS-CoV-2 (infection or vaccination) were included into this study. Patient characteristics are described in the Supplementary Materials in Table S1.
Recruitment	Participants with different numbers of exposure to SARS-CoV-2 (infection or vaccination) were included into this study. No compensation for participation in the study was paid. Samples were included based on vaccination and/or infection status of participants. Participants provided information on vaccination status and previous infection. Sample selection might be biased due to limited sample size, however this bias should not influence the major conclusion of the study as relative changes of neutralizing antibodies within each individual patient were calculated for different SARS-CoV-2 variants.
Ethics oversight	The ethics committee (EC) of the Medical University of Innsbruck has approved the study with EC numbers: 1100/2020, 1111/2020, 1330/2020, 1064/2021, 1093/2021, 1168/2021, 1191/2021, and 1197/2021. Informed consent has been obtained from study participants.

Note that full information on the approval of the study protocol must also be provided in the manuscript.

Field-specific reporting

Please select the one below that is the best fit for your research. If you are not sure, read the appropriate sections before making your selection.

☒ Life sciences ☐ Behavioural & social sciences ☐ Ecological, evolutionary & environmental sciences

For a reference copy of the document with all sections, see nature.com/documents/nr-reporting-summary-flat.pdf

Life sciences study design

All studies must disclose on these points even when the disclosure is negative.

Sample size	No sample size calculation was performed. We chose sample sizes based in previous studies. Additionally, we performed map diagnosis as described in the Supplementary Materials to ensure robustness of the generated antigenic maps.
Data exclusions	No data were excluded.
Replication	We previously decribed the reproducibility of the here used neutralization assay. Each sample was analyzed once in the assay. For antigenic mapping, robustness of maps has been analyzed as detailed in the Supplementary Methods. For details regarding map diagnosis see Supplementary Materials Figures S3-S10, S14-18.
Randomization	Not applicable as participants were allocated to the different groups due to the number of exposures with SARS-CoV-2
Blinding	No blinding was performed. Number of infected cells in the neutralization assay were automatically counted using an immunospot reader. Manual quality control to remove fibres etc. has been done blinded.

Reporting for specific materials, systems and methods

We require information from authors about some types of materials, experimental systems and methods used in many studies. Here, indicate whether each material, system or method listed is relevant to your study. If you are not sure if a list item applies to your research, read the appropriate section before selecting a response.

Materials & experimental systems

n/a	Involved in the study
☐	☒ Antibodies
☐	☒ Eukaryotic cell lines
☒	☐ Palaeontology and archaeology
☒	☐ Animals and other organisms
☒	☐ Clinical data
☒	☐ Dual use research of concern

Methods

n/a	Involved in the study
☒	☐ ChIP-seq
☒	☐ Flow cytometry
☒	☐ MRI-based neuroimaging

Antibodies

Antibodies used	Polyclonal Alexa Fluor Plus 488-conjugated goat anti-human IgG secondary antibody (1:1000 diluted, Ref. A48276, Invitrogen, Thermo Fisher Scientific, Vienna, Austria)
Validation	No antibody validation has been performed.

Eukaryotic cell lines

Policy information about cell lines and Sex and Gender in Research

Cell line source(s)	In-house generated Vero (African green monkey kidny cell line) derivate stably overexpressing ACE2 and TMPRSS2
Authentication	no cell line authentication
Mycoplasma contamination	Cells were tested negative for mycoplasma
Commonly misidentified lines (See ICLAC register)	No commonly missidentified cell line has been used for this study.
